# Multiple model trajectory poisson multi-bernoulli mixtures filter for tracking multiple maneuvering objects

**DOI:** 10.1038/s41598-025-28096-1

**Published:** 2025-12-17

**Authors:** Ibrahim Salim, Nermeen Okasha, Mohamed Barbary, Wagdy Anis

**Affiliations:** 1 Electronics Engineering and Electrical Communications, Ain Shams University, Cairo, Egypt; 2 Electronics and Communications Engineering, Modern Academy for Engineering and Technology, Cairo, Egypt; 3 Department of Electrical Engineering, Alexandria University, Alexandria, Egypt

**Keywords:** Multi-Object tracking, Multiple-Model, PMBM, Jump markov system, Trajectory, Mathematics and computing, Electrical and electronic engineering

## Abstract

Multi-object tracking (MOT) in cluttered and dynamic environments remains challenging, especially for maneuvering objects. While trajectory-based random finite set (RFS) filters provide principled solutions for trajectory estimation, they typically rely on a single motion model, limiting their adaptability. To address this gap, we propose the Multiple-Model Trajectory Poisson Multi-Bernoulli Mixture (MM-TPMBM) filter, which integrates jump Markov system (JMS) dynamics within the trajectory RFS framework. The filter enables closed-form Bayesian recursion for joint trajectory estimation and motion model switching. We derive its prediction and update equations, implement it using Gaussian mixtures for computational efficiency, and evaluate performance against benchmark filters (MM-PMBM, TPMBM, δ-GLMB) via Monte Carlo simulations. Results demonstrate that the MM-TPMBM filter achieves superior accuracy in trajectory estimation, localization, and cardinality, reducing the generalized optimal sub-pattern assignment (GOSPA) error by up to 24% and cardinality error by up to 52% compared to state-of-the-art methods, validating its robustness in complex tracking scenarios.

## Introduction

Multi-object tracking (MOT) plays a pivotal role in a wide range of applications^1–6^, including autonomous navigation, surveillance, and radar systems. MOT is a critical capability in modern intelligent systems, enabling real-time awareness, decision-making, and safety across a range of high-stakes environments. In autonomous vehicles, MOT allows the system to continuously detect and predict the behavior of surrounding cars, pedestrians, cyclists, and obstacles, helping prevent accidents and ensuring smooth, safe navigation. Within airport airfields, MOT plays a vital role in managing the complex movement of aircraft, ground support vehicles, and personnel particularly in low-visibility or high-traffic conditions, thus preventing collisions and maintaining operational efficiency. In state defense systems, MOT becomes indispensable for detecting and intercepting foreign missiles, drone swarms, and even unidentified flying objects by tracking multiple high-speed threats simultaneously^5–7^, prioritizing them based on threat, and coordinating responses across layered defense platforms^8–10^. The integration of MOT with advanced sensors and AI models enables predictive tracking, enhances system resilience, and supports critical real-time decisions that safeguard lives, infrastructure, and national security^11^. Technically, the core challenge lies in accurately estimating the states and trajectories of multiple objects in the presence of measurement noise, clutter, and missed detections. Over the years, several probabilistic frameworks have been proposed to address this challenge, with varying degrees of success and scalability^12–15^.

### Background

Traditional approaches such as Multiple Hypothesis Tracking (MHT)^16–18^ and Joint Probabilistic Data Association (JPDA)^19,20^ have long served as the backbone of MOT systems. JPDA offers a probabilistic mechanism for associating measurements to objects, while MHT maintains multiple competing hypotheses over time to resolve ambiguities. Despite their historical significance, these methods often suffer from scalability issues and heuristic approximations, particularly in dense or cluttered environments. To address these limitations, the Random Finite Set (RFS) framework was developed, providing a mathematically principled approach to jointly estimating both the number and states of multiple objects. Within this framework, filters like the Probability Hypothesis Density (PHD)^21^, Cardinalized PHD (CPHD)^22^, and the Poisson Multi-Bernoulli Mixture (PMBM)^23,24^ have demonstrated strong performance. In particular, the PMBM filter offers a closed-form Bayesian solution to the multi-object filtering problem and provides an elegant mechanism for modeling undetected objects using a Poisson point process. This advantage allows it to efficiently handle high-clutter environments and varying object counts.

### Research gap

Although the PMBM filter has demonstrated strong performance in multi-object tracking, it primarily focuses on estimating object sets at each time step without preserving trajectory information, limiting its effectiveness in applications that require temporal coherence. Recent advances in trajectory-based RFS filters such as TPHD, TCPHD, TMB, TPMB, and TPMBM^25–31^ address this by modeling the evolution of object states over time. However, these filters generally assume a single motion model, which is insufficient for tracking highly maneuverable objects in dynamic environments. The integration of multiple motion models (MM) via approaches such as the jump Markov system (JMS) has shown promise but remains underexplored within the trajectory RFS framework. Consequently, there exists a significant gap in the development of closed-form, computationally feasible multi-object tracking filters that can simultaneously accommodate both trajectory estimation and multiple motion dynamics.

### Problem statement and motivation

The core challenge in the development of a trajectory-based filtering framework is making it 1) capable of accurately tracking maneuvering objects while maintaining 2) resilience to clutter and 3) computational efficiency. In many practical scenarios, objects exhibit abrupt changes in motion, such as evasive maneuvers or mode switching that cannot be captured by single-model assumptions. Moreover, trajectory estimation is increasingly important for decision-making tasks that rely on temporal object behavior rather than instantaneous positions.

Addressing the aforementioned research gap is the main objective of this paper’s proposed MM-TPMBM filter, which integrates JMS-based dynamics into the trajectory RFS formalism, offering a principled, closed-form, and computationally efficient solution to robust multi-object trajectory tracking. The Multiple Model (MM) approach, particularly using the JMS framework^32–34^, addresses this challenge by introducing a hidden motion model variable that evolves according to a Finite State Markov Chain (FSMC)^35^. This motivates the development of a Multiple-Model Trajectory PMBM (MM-TPMBM) filter, which integrates multiple motion models and trajectory estimation in a unified framework. This integration enables the filter to adapt to varying motion patterns while preserving the structural advantages of trajectory estimation.

The limitations of MM-PMBM and label-based trajectory construction highlight the need for a unified filtering framework that combines trajectory-centric estimation with multiple motion model adaptability. The proposed MM-TPMBM filter enables direct estimation of maneuvering object trajectories while preserving the closed-form structure and scalability of TPMBM. By building on the strengths of CPHD, TPHD, and PMBM-based filters^36–38^, MM-TPMBM offers a principled and robust solution for multi-object tracking in cluttered and dynamic environments.

### Key contributions

This paper presents the MM-TPMBM filter and offers several key contributions:

- Derive a closed-form prediction and update equations for trajectory filtering under a multiple model framework, extending the theoretical foundation of TPMBM to accommodate JMS dynamics.
- Implement the filter using Gaussian mixture techniques, ensuring computational tractability and facilitating real-world deployment.
- Conduct a comprehensive performance evaluation through Monte Carlo simulations, comparing MM-TPMBM against benchmark filters MM-PMBM, TPMBM, and δ-GLMB filters.
- Demonstrate that MM-TPMBM achieves superior performance in trajectory accuracy, localization precision, and cardinality estimation.

### Key limitations

While the MM-TPMBM filter offers notable advancements, it is not without limitations:

- Increased Computational Cost: Integrating multiple motion models leads to a higher number of Gaussian components, which increases the computational demands of the filter.
- Sensitivity to Model Specification: The filter’s performance is highly dependent on the accurate specification of motion models and their transition probabilities, which can be difficult to determine in practical applications.
- Hypothesis Management: In scenarios with many objects or long tracking periods, the rapid growth of the hypothesis space requires aggressive pruning, which can potentially degrade estimation accuracy.
- Validation Scope: The current validation is based on simulations; real-time implementation and performance in large-scale, real-world implementation have not yet been investigated.

### Paper organization

The article is structured as follows: A comprehensive literature review is outlined in Sect.  “Literature Review”. Section  “Problem Formulation and Modelling” presents the problem formulation and modeling of the problem. Section  “The Proposed MM-TPMBM Filter” examines the JMS model and the proposed MM-TPMBM filter, including the filter recursion process. Section  “Discussion” outlines the comprehensive implementations of the MM-TPMBM filter utilizing the GM approach. The simulation results are given in Section  “The Implementation”, and the conclusions are provided in Sect.  “Conclusion”.

## Literature review

MOT field has evolved significantly over the past decades, driven by the need to reliably estimate and maintain the states of multiple dynamic objects in cluttered and uncertain environments. Early approaches primarily focused on probabilistic data association methods, which provided foundational insights but faced challenges in scalability and flexibility. These limitations motivated the development of RFS-based methods, which introduced a rigorous mathematical framework for handling uncertainty in both object number and data association. More recently, trajectory-based extensions and multiple-model formulations have emerged^39,40^, offering more robust solutions for complex and maneuvering scenarios.

### Classical approaches: JPDA and MHT

The Joint Probabilistic Data Association (JPDA) filter models the association of measurements to objects using probabilistic weights, assuming a fixed number of objects. While effective in moderately cluttered environments, JPDA struggles with scalability and often relies on heuristic gating and pruning. Multiple Hypothesis Tracking (MHT), on the other hand, maintains a tree of hypotheses over time, offering greater flexibility but at the cost of significant computational overhead. Both methods are limited by their reliance on explicit data association and their inability to represent uncertainty in the number of objects in a principled way.

### Development of RFS-based filters

To address these limitations, RFS^41,42^ theory was introduced as a mathematically rigorous framework for multi-object tracking. The PHD filter approximates the multi-object posterior using its first-order moment, enabling efficient estimation without explicit data association^43^. However, it does not account for object count uncertainty.

The Cardinalized PHD (CPHD) filter extends the PHD framework by incorporating cardinality information, thereby improving performance in scenarios with fluctuating object numbers. Both PHD and CPHD filters are computationally efficient and have been widely adopted, though they do not maintain object identities or trajectories.

The Generalized Labeled Multi-Bernoulli (GLMB)^44,45^ filter represents a major advancement, introducing labeled RFSs to preserve object identities over time. It offers closed-form solutions under certain assumptions and has demonstrated strong performance in cluttered environments. These developments laid the groundwork for trajectory-centric filtering.

### Trajectory-based RFS filters

Recognizing the need for temporally coherent tracking, researchers have extended RFS theory to operate directly on trajectories. The Trajectory PHD (TPHD) and Trajectory CPHD (TCPHD) filters adapt the PHD and CPHD formulations to estimate entire trajectories rather than instantaneous states. These filters provide a principled way to model motion over time, though they inherit the limitations of their base formulations in terms of identity preservation.

More advanced filters such as the Trajectory MB (TMB), Trajectory PMB (TPMB), and Trajectory PMBM (TPMBM) offer closed-form solutions and improved performance in low detection probability scenarios. Among these, TPMBM stands out for its ability to estimate trajectory sets directly and has been successfully implemented in both point and extended object tracking. Its closed-form recursions for trajectory filtering make it a compelling candidate for real-world applications.

### Multiple model filtering and the MM-PMBM framework

In dynamic environments, relying on a single motion model can lead to degraded tracking performance. The Multiple Model (MM) approach, particularly JMS, introduces a motion model parameter into each object state, evolving over time via a Finite State Markov Chain (FSMC). The MM-PMBM filter integrates JMS dynamics into the PMBM framework, enabling adaptive tracking of maneuvering objects^46,47^. However, it remains object-centric and does not extend naturally to trajectory estimation.

## Problem formulation and modelling

This section introduces the fundamentals of the RFS trajectory. Then, we outline the modeling assumptions employed in this study. Further, we briefly introduce the set of trajectories framework for the transition and measurement model. Lastly, it provides an overview of the PMBM filter.

### Modelling assumptions

At time step $t$, existing objects ${x}_{t-1}$ survive to the next step with probability ${p}_{s}\left({x}_{t-1}\right)$ or fail to exist with probability $1-{p}_{s}\left({x}_{t-1}\right)$. The Markov state transition function of an object, if it exists, is ${f}_{k}\left(\cdot |{x}_{t-1}\right)$. The discovered objects ${x}_{t}$ at each time step do not depend on any other objects that have survived and adhere to a PPP with a birth intensity of ${\mu }_{t}^{b}\left({x}_{t}\right)$. The measurement set for objects has been split into two segments: the first segment includes real measurement sequences produced by sensors that detect a single object, represented as PPPs with measurement likelihoods of ${l}_{t}\left({z}_{t}|{x}_{t}\right)$ produced by each ${x}_{t}\in {X}^{{d}_{t}}$ and with a Poisson intensity of ${\mu }_{t}\left({x}_{t}\right)$. While the other segment includes measurements of noise, which is modeled as Poisson RFS and ${\gamma }_{t}^{C}$ rate and a spatial distribution $C\left(z\right)$. The intensity of the clutter, which is also a Poisson distribution, is ${\mu }_{t}^{C}\left({z}_{t}\right)={\gamma }_{t}^{C}C\left(z\right)$.

### Trajectories’ RFSs

Let $\mathcal{X}$ denote the state space of a single object, e.g., $\mathcal{X}={\mathbb{R}}^{4}$, where $\mathcal{X}$ is a two-dimensional state representing location and velocity. We employ the model of trajectory state delineated in^48^, wherein the trajectory state is denoted as a set $\mathsf{X}=\left(\beta ,\epsilon ,{x}_{\beta :\epsilon }\right)$, for the trajectory’s birth $\beta$ denotes the discrete and for the trajectory’s end $\epsilon$ indicates the discrete time. If $t$ represents the current time, then $\epsilon =t$ indicates that the trajectory is alive; ${x}_{\beta :\epsilon }$ is defined in relation to *β* and $\epsilon$, the state sequence is

1 $$\begin{array}{c}{x}_{\beta },{x}_{\beta +1}, \dots ,{x}_{\epsilon -1}, {x}_{\epsilon }\end{array}$$

where ${x}_{t}\in \mathcal{X}$ for all $t\in \left\{\beta ,\dots ,\epsilon \right\}$. It results in a trajectory with length $l=\epsilon -\beta +1$ time steps. At time $t$, the state space of the trajectory is^49^

2 $$\begin{array}{c}{T}_{t}={\uplus }_{\left(\beta ,\epsilon \right)\in {I}_{t}}\left\{\beta \right\}\times \left\{\epsilon \right\}\times {\mathcal{X}}^{\epsilon -\beta +1}\end{array}$$

where $\uplus$ denotes disjoint set union, ${I}_{t}=\left\{\left(\beta ,\epsilon \right):0\le \beta \le \epsilon \le t\right\}$ and the Cartesian products of $\mathcal{X}$ are represented by ${\mathcal{X}}^{l}$. The following is the factorization of the trajectory state density of ${\mathsf{X}}_{t}$, which is derived from measurements until and including time $t\ge t-1$,

3 $$\begin{array}{c}{p}_{t|t-1}\left(\mathsf{X}\right)={p}_{t|t-1}\left({x}_{\beta :\epsilon }|\beta ,\epsilon \right){P}_{t|t-1}\left(\beta ,\epsilon \right)\end{array}$$

where, if $\epsilon <\beta$, then ${P}_{t|t-1}\left(\beta ,\epsilon \right)$ is zero. The following is how integration for single trajectory densities is carried out^49^

4 $$\begin{array}{c}\int p\left(\mathsf{X}\right)d\mathsf{X}= \sum_{\beta ,\epsilon }\left[\int \dots \int p\left({x}_{\beta },\dots ,{x}_{\epsilon }|\beta ,\epsilon \right)d{x}_{\beta }\dots d{x}_{\epsilon }\right]P\left(\beta ,\epsilon \right)\end{array}$$

where $\mathcal{F}\left({{\varvec{T}}}_{t}\right)$ denotes a set of all finite subsets of ${{\varvec{T}}}_{t}$, ${{\varvec{X}}}_{t}\in \mathcal{F}\left({{\varvec{T}}}_{t}\right),$ represents a set of trajectories. In a set of trajectories, the set integral of a real-valued function $g\left({{\varvec{X}}}_{t}\right)$ is

5 $$\int {g(X_{t} )\delta } X_{t} \triangleq g(\emptyset ) + \sum\limits_{n = 1}^{\infty } {\frac{1}{n!}\int { \cdots \int {g(\{ X_{t}^{1} , \ldots ,} } } X_{t}^{n} \} )dX_{t}^{1} \ldots dX_{t}^{n}$$

The Poisson RFS and the Bernoulli RFS are two fundamental components of RFSs-based MOT. The density of a trajectory Poisson RFS is:

6 $$f^{ppp} (X) = e^{{ - \int {D(X_{k - 1} )dX_{k - 1} } }} \prod\limits_{X \in X} {\mu (X)}$$

where the trajectory state space ${{\varvec{T}}}_{t}$ defines the trajectory Poisson RFS intensity $D\left(\cdot \right)$. The trajectory of a Bernoulli RFS has density,

7 $$\begin{array}{c}{f}^{ber}\left({\varvec{X}}\right)=\left\{\begin{array}{c}1-r, X=\varnothing \\ r f\left(\mathsf{X}\right), X=\left\{\mathsf{X}\right\}\\ 0, otherwise\end{array}\right.\end{array}$$

where $f\left(\cdot \right)$ is a single trajectory density. Combining $f\left(\cdot \right)$ and $r$ provides the probability that the object state existed at a specific time or that the object trajectory was in a specific location at a given time.

### Transition model

The standard transition model of multi-object employs a Poisson RFS to describe the object’s birth at time $t$, we describe the birth intensity as

8 $$\begin{array}{c}{D}_{t}^{b}\left(\mathsf{X}\right)={D}_{t}^{b,x}\left({x}_{\beta :\epsilon }|\beta ,\epsilon \right){\Delta }_{t}\left(\epsilon \right) {\Delta }_{t}\left(\beta \right)\end{array}$$

9 $$\begin{array}{c}{D}_{t}^{b,x}\left({x}_{t:t}|t,t\right)={D}_{t}^{b}\left({x}_{t}\right)\end{array}$$

where $\Delta \left(\cdot \right)$ denotes Kronecker delta function. All the trajectories set, ${{\varvec{X}}}_{t}$ contains all trajectories where $0\le \beta \le \epsilon \le t$. The survival probability at time $t$ as a function of trajectories is defined as follows,

10 $$\begin{array}{c}{p}_{S,t}\left(\mathsf{X}\right)={p}_{S}\left({x}_{t}\right){\Delta }_{t}\left(\epsilon \right)\end{array}$$

The problem formulation provides the trajectories’ transition density. The Bernoulli RFS transition density in the absence of birth is

11 $$\begin{array}{c}{f}_{t|t-1}\left({{\varvec{X}}}_{t}|{{\varvec{X}}}_{t-1}\right)=\left\{\begin{array}{c} 1, {{\varvec{X}}}_{t-1}=\varnothing , {{\varvec{X}}}_{t}=\varnothing \\ \pi \left({\mathsf{X}}_{t}|{\mathsf{X}}_{t-1}\right), {{\varvec{X}}}_{t-1}=\left\{{\mathsf{X}}_{t-1}\right\}\\ 0, otherwise\end{array}\right., X=\left\{\mathsf{X}\right\}\end{array}$$

12 $$\begin{array}{c}\pi \left({\mathsf{X}}_{t}|{\mathsf{X}}_{t-1}\right)={\pi }^{x}\left({x}_{\beta :\epsilon }|\beta ,\epsilon ,{\mathsf{X}}_{t-1}\right){\pi }^{\epsilon }\left(\beta |\epsilon ,{\mathsf{X}}_{t-1}\right){\Delta }_{\beta ,t-1}\left(\beta \right)\end{array}$$

where $\pi \left({\mathsf{X}}_{t}|{\mathsf{X}}_{t-1}\right)$ is single object transition density, and

13 $$\begin{array}{c}{\pi }^{\epsilon }\left(\epsilon |\beta ,{\mathsf{X}}_{t-1}\right)=\left\{\begin{array}{c}1, \epsilon ={\epsilon }_{t-1}<t-1\\ 1-{p}_{S,t-1}\left({\mathsf{X}}_{t-1}\right), \epsilon ={\epsilon }_{t-1}=t-1\\ {p}_{S,t-1}\left({\mathsf{X}}_{t-1}\right), \epsilon ={\epsilon }_{t-1}+1=t\\ 0, otherwise \end{array}\right.\end{array}$$

14 $$\begin{array}{c}{\pi }^{x}\left({x}_{k,\beta :\epsilon }|\beta ,\epsilon ,{\mathsf{X}}_{t-1}\right)=\left\{\begin{array}{c}{\delta }_{{x}_{t-1,\beta :\epsilon }}\left({x}_{t,\beta :\epsilon }\right), \epsilon ={\epsilon }_{t-1}\\ {\pi }^{x}\left({x}_{t,\epsilon }|{x}_{t-1,\epsilon }\right){{\delta }_{{x}_{t-1,{\beta }_{t-1}:{\epsilon }_{t-1}}}}_{ }\left({x}_{t,\beta :\epsilon -1}\right), \epsilon ={\epsilon }_{t-1}+1\end{array}\right.\end{array}$$

According to this model, the survival probability controls whether the trajectory terminates or continues for one more step. Combining the birth process with the set of trajectories formed from the prior set of trajectories generates the predicted set of trajectories.

### The PMBM filter

The density of PMBM RFS is structurally described as a mixture of the Poisson RFS density ${f}^{p}\left(\cdot \right)$ and MBM RFS density ${f}^{mbm}\left(\cdot \right)$ given by

15 $$\begin{array}{c}f\left(\mathbf{X}\right)={\sum }_{{\text{X}}^{u}\uplus {\text{X}}^{d}=\mathbf{X}}{f}^{p}\left({\mathbf{X}}^{u}\right) {f}^{mbm}\left({\mathbf{X}}^{d}\right)\end{array}$$

where ${\mathbf{X}}^{d}$ and ${\mathbf{X}}^{u}$ represent the potentially detected objects and undetected objects, respectively. Furthermore, a PMB RFS density will degenerate from the PMBM RFS density with a single global hypothesis. The MBM has ${n}_{t|t-1}$ Bernoulli components (potential objects), each with ${h}_{{n}_{t|t-1}}^{i}$ local hypotheses. For each Bernoulli $i>0$, there exists a local hypothesis ${a}^{i}\in \{1,\dots ,{h}_{{n}_{t|t-1}}^{i}\}$. The *i-th* Bernoulli, for $i\ge 0$, or the clutter, for $i=0$, are associated to a certain set of subsets of the measurement set by each ${a}^{i}$, which is an index. A global hypothesis is represented by $a=\left({a}^{0}, {a}^{1},\dots ,{a}^{{n}_{t|t-1}}\right)\in {\mathcal{A}}_{t|t-1}$ where ${\mathcal{A}}_{t|t-1}$ is the global hypotheses set and ${a}^{0}$ is the local hypothesis of clutter. ${\omega }_{t|t-1}^{a}$ represents the weight of global hypothesis $a$,

16 $$\begin{array}{c}{\omega }_{t|t-1}^{a}\propto {\prod }_{i=0}^{{n}_{t|t-1}}{\omega }_{t|t-1}^{i,{a}^{i}}\end{array}$$

where ${\omega }_{t|t-1}^{i,{a}^{i}}$ denotes the weight of the *i-th* Bernoulli component, or clutter if $i=0$, and

17 $$\begin{array}{c}{\sum }_{a\in {\mathcal{A}}_{t|t-1}}{\omega }_{t|t-1}^{a}=1\end{array}$$

**Fig. 1:**
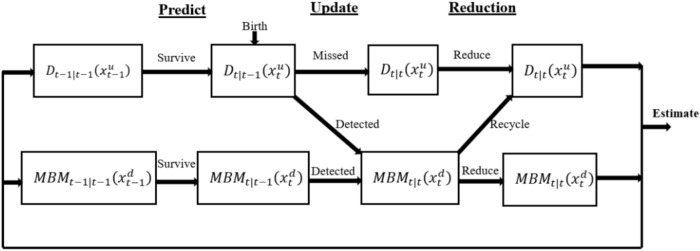
Schematic representation of the PMBM filter, the update step incorporates the measurement information. The recycling process allows to retain information about distributions not associated with any measurements.

PMBM filters with PPP clutter differ in that global hypotheses and weights explicitly account for clutter^24,50,51^, indicated by $i=0$. Thus, ${\sum }_{i=1}^{{n}_{t|t-1}}{h}_{{n}_{t|t-1}}^{i}$ is the sum of the Bernoulli densities for all global hypotheses, and the quantity of multi-Bernoulli densities is denoted as ${|\mathcal{A}}_{t|t-1}|$ , represented by the cardinality of ${\mathcal{A}}_{t|t-1}$^52^. The PMBM filter recursive processes are illustrated in Fig. 1, dependent on the measurement set ${{\varvec{Z}}}_{1:t}$ , are outlined as follows:

*Prediction Step:* Both the undetected and potentially detected objects’ posterior densities, denoted as ${f}^{p}\left({{\varvec{X}}}^{u}\right)$ and ${f}^{mbm}\left({{\varvec{X}}}^{d}\right)$, can be predicted separately. Let ${D}_{t-1}\left(x\right)$ be the Poisson intensity at time $t-1$ thus, at time $t$, the predicted intensity is

18 $$\begin{array}{c}{D}_{t|t-1}\left(x\right)={D}_{t}^{b}\left(x\right)+\langle {f}_{t|t-1}\left(x|\cdot \right){p}_{S,t}\left(\cdot \right),{D}_{t-1}\left(\cdot \right)\rangle \end{array}$$

where ${D}_{t}^{b}\left(x\right)$ represents the birth model intensity, the state transition function is represented by ${f}_{t|t-1}\left(x|\cdot \right)$, and the survival probability is denoted by ${p}_{S,t}\left(\cdot \right)$. Additionally, $\langle \cdot , \cdot \rangle$ represents the inner product. The predicted Bernoulli density for each Bernoulli component in the MBM density can be calculated as follows:

19 $$\begin{array}{c}{r}_{t|t-1}^{i,{a}^{i}}= {r}_{t-1}^{i,{a}^{i}}\langle {p}_{t-1}^{i,{a}^{i}}\left(\cdot \right), {p}_{S,t}\left(\cdot \right)\rangle \end{array}$$

20 $$\begin{array}{c}{p}_{t|t-1}^{i,{a}^{i}}\left(x\right)\propto \langle {f}_{t|t-1}\left(x|\cdot \right){p}_{S,t}\left(\cdot \right),{p}_{t-1}^{i,{a}^{i}}\left(\cdot \right)\rangle \end{array}$$

where ${r}_{t|t-1}^{i,{a}^{i}}$ and ${p}_{t|t-1}^{i,{a}^{i}}\left(x\right)$ represent the probability of predicted existence and probability density of the global hypothesis’s *i-th* Bernoulli component, respectively. In addition, the weight of hypothesis remains constant; ${\omega }_{t|t-1}^{i,{a}^{i}}={\omega }_{t-1}^{i,{a}^{i}}$.

*Update Step:* The posterior PDF is transformed from the predicted posterior PDF at time $t$ using the Bayes rule. The intensity of the PPP is

21 $$D_{t|t} \, (x) = f(\emptyset |x)D_{t|t - 1} (x)$$

The update of undetected objects matches with the PHD filter update in scenarios empty of measurements. The update of PPP intensity for undetected objects that remain undetected is performed by

22 $$\begin{array}{c}{D}_{t}\left(x\right)=\left(1-{p}_{D,t}\left(x\right)\right){D}_{t|t-1}\left(x\right)\end{array}$$

where ${p}_{D,t}\left(\cdot \right)$ denotes the detection probability. Two primary categories of detected objects are new Bernoulli components and legacy Bernoulli components. First, let’s take a look at the new Bernoulli components that the Poisson components have produced,

23 $$\begin{array}{c}{r}_{t}^{p}\left(z\right)={s}_{t}\left(z\right)/{\rho }_{t}^{p}\left(z\right)\end{array}$$

24 $$\begin{array}{c}{p}_{t\left(x|z\right)}^{p}={p}_{D,t}\left(x\right){l}_{t}\left(z|x\right){D}_{t|t-1}\left(x\right)/{s}_{t}\left(z\right)\end{array}$$

where,

25 $$\begin{array}{c}{s}_{t\left(z\right)}=\langle {l}_{t\left(z|\cdot \right)}{p}_{D,t}\left(\cdot \right),{D}_{t|t-1\left(x\right)}\left(\cdot \right)\rangle \end{array}$$

26 $$\begin{array}{c}{\rho }_{t\left(z\right)}^{p}={s}_{t}\left(z\right)+{c}_{t}\left(z\right)\end{array}$$

where $c\left(\cdot \right)$ represents the clutter intensity, and ${l}_{t}\left(z|x\right)$ is the measurement likelihood function. Next, we will review the legacy Bernoulli components. The update process of legacy Bernoulli components can be divided based on whether they are updated by measurements: misdetection and measurement-based updates. For misdetection cases, the posterior PDF corresponds to the predicted posterior PDF,

27 $$\begin{array}{c}{\omega }_{t}^{i,{a}^{i},0}={\omega }_{t|t-1}^{i,{a}^{i}}\left(1-{p}_{D,t}\left(x\right){r}_{t|t-1}^{i,{a}^{i}}\right)\end{array}$$

28 $$\begin{array}{c}{r}_{t}^{i,{a}^{i},0}={r}_{t|t-1}^{i,{a}^{i}}\left(1-{p}_{D,t}\left(x\right)\right)/\left(1-{p}_{D,t}\left(x\right){r}_{t|t-1}^{i,{a}^{i}}\right)\end{array}$$

On the other hand, for the measurement-based update, the existence probability of a measurement-produced Bernoulli component is set to 1, and

29 $$\begin{array}{c}{\omega }_{t}^{i,{a}^{i}}={\omega }_{t|t-1}^{i,{a}^{i}}{r}_{t|t-1}^{i,{a}^{i}}\langle {P}_{D,t}{l}_{t}\left(z|\cdot \right),{P}_{t|t-1}^{i,{a}^{i}}\left(\cdot \right)\rangle \end{array}$$

30 $$\begin{array}{c}{P}_{t}^{i,{a}^{i}}\left(x|z\right)\propto {P}_{D,t}\left(x\right){l}_{t}\left(z|x\right){P}_{t|t-1}^{i,{a}^{i}}\left(x\right)\end{array}$$

At this point, only the single-object hypotheses have been generated. Subsequently, new global hypotheses are generated by examining all potential combinations of single-object hypotheses. A cost matrix is built using the weights of new hypothesis in both Eqs. (27) and (29) and forms the basis of Murty’s algorithm. Consult^53^ for the detailed implementation procedures.

## The proposed MM-TPMBM filter

This section introduces the proposed Multi-Model Trajectory Poisson Multi-Bernoulli Mixture (MM-TPMBM) filter, designed to enhance object tracking capabilities for maneuvering objects. Standard tracking filters often rely on a single motion model, which can lead to performance degradation or track loss when an object’s dynamics change unexpectedly. To address this limitation, our proposed approach integrates JMS into the Trajectory PMBM (TPMBM) framework. This integration allows each potential trajectory to be associated with a set of motion models, with the probability of each model evolving over time according to a Markov chain. By dynamically switching between kinematic models, the MM-TPMBM filter provides a more robust and accurate representation of the object’s state.

### Jump markov system

The variable $\xi \in {\mathbb{M}}$ represents the motion model of a manoeuvring object, which is associated with the state variable $x\in {\mathbb{X}}$, resulting in the augmented state vector represented as $\widetilde{x} =\left(x,\xi \right)\in {\mathbb{X}}\times {\mathbb{M}}$. Concurrently, the multi-object state can be defined as follows:

31 $$\begin{array}{c}\widetilde{{\varvec{X}}}=\left\{{\widetilde{x}}_{1},\dots ,{\widetilde{x}}_{2}\right\}=\left\{\left({x}_{1},{\xi }_{1}\right),\dots ,\left({x}_{n},{\xi }_{n}\right)\right\}\end{array}$$

Each object’s motion models change in accordance with a finite-state Markov chain under JMS. The transition matrix $\Pi$ is formed in a linear JMS where the transition probabilities between any two models $\eta (\xi |\xi^{\prime})$ remain constant. However, in order to find the fundamental state’s probability density function, variable $\xi$ can be removed as an unnecessary variable,

32 $$\begin{array}{c}f\left(x\right)={\sum }_{\xi \in {\mathbb{M}}}\eta \left(\xi \right)f\left(x|\xi \right)\end{array}$$

where $f\left(x|\xi \right)$ and $\eta \left(\xi \right)$ respectively denote conditional density function and model probability. Conversely, the inner product of any two functions $f\left(\cdot \right)$ and $g\left(\cdot \right)$ is defined as:

33 $$\begin{array}{c}\langle f\left(\cdot ,\cdot \right),g\left(\cdot ,\cdot \right)\rangle ={\sum }_{\xi \in {\mathbb{M}}}\int f\left(x,\xi \right)g\left(x,\xi \right)dx\end{array}$$

For the augmented single-object state, the following functions are defined: transition density and measurement likelihood,

34 $$\begin{array}{c}{f}_{t|t-1}\left({x}_{t},{\xi }_{t}|{x}_{t-1},{\xi }_{t-1}\right)={f}_{t|t-1}\left({x}_{t}|{x}_{t-1},{\xi }_{t-1}\right){\eta }_{t|t-1}\left({\xi }_{t}|{\xi }_{t-1}\right)\end{array}$$

35 $$\begin{array}{c}{l}_{t}\left(z|x,\xi \right)={l}_{t}\left(z|x\right)\end{array}$$

where ${f}_{t|t-1}\left({x}_{t}|{x}_{t-1},{\xi }_{t-1}\right)$ and ${\eta }_{t|t-1}\left({\xi }_{t}|{\xi }_{t-1}\right)$ represent the state transition density and the density of the model transition, respectively. As demonstrated in (42), the motion model is usually independent to the measurement likelihood function.

### MM-TPMBM filter

This subsection’s description closely aligns with the standard PMBM filter. For point objects, we expand the trajectory PMBM (TPMBM) filter’s closed-form filtering recursion to MM-TPMBM filter, the density of the trajectory PMBM is:

36 $$\begin{array}{c}{f}_{t|t-1}\left({{\varvec{X}}}_{t}\right)= \sum_{{{\varvec{X}}}_{t}^{u}\uplus {{\varvec{X}}}_{t}^{d}=\mathbf{X}}{f}_{t|t-1}^{\text{PPP}}({{\varvec{X}}}_{t}^{u})\sum_{a\in {\mathcal{A}}_{t|t-1}}{\omega }_{t|t-1}^{a} {f}_{t|t-1}^{a}\left({{\varvec{X}}}_{t}^{d}\right)\end{array}$$

the density of a Poisson Random Finite Set is

37 $$\begin{array}{c}{f}_{t|t-1}^{\text{PPP}}\left({{\varvec{X}}}_{t}^{u}\right)= {e}^{-\int {D}_{t|t-1}^{u}\left({\mathsf{X}}_{{\varvec{t}}-1}\right)d{\mathsf{X}}_{{\varvec{t}}-1}}\prod_{\mathsf{X}\in {{\varvec{X}}}_{t}^{u}}{\mu }_{t|t-1}^{u}\left(\mathsf{X}\right)\end{array}$$

the density of a MBM RFS is

38 $$\begin{array}{c}{f}_{t|t-1}^{a}\left({{\varvec{X}}}_{t}^{d}\right)= \sum_{{\uplus }_{{i}_{t-1} \in { \mathcal{T}}_{t|t-1}}{{\varvec{X}}}_{t}^{{i}_{t-1} }={\mathbf{X}}_{t}^{{\varvec{d}}}} \prod_{i\in { \mathcal{T}}_{t|t-1}}{f}_{t|t-1}^{i,{a}^{i}}\left({{\varvec{X}}}_{t}^{i}\right)\end{array}$$

the set of trajectories ${{\varvec{X}}}_{t}$ is formed by the independent union of a Poisson RFS ${{\varvec{X}}}_{t}^{u}$ with intensity ${D}_{t|t-1}^{u}$ and a MBM RFS ${{\varvec{X}}}_{t}^{d}$ with Bernoulli parameters ${r}_{t|t-1}^{i,{a}^{i}}$ and ${f}_{t|t-1}^{i,{a}^{i}}$. In the Poisson RFS, trajectories are defined as not detected if no measurements have been associated with them, even though they are expected to exist. For the MBM,${\mathcal{T}}_{t|t-1}$ is a track table with ${n}_{t|t-1}$ tracks, for every global hypothesis $a$ and every track $i\in { \mathcal{T}}_{t|t-1}$, there is a global data association hypothesis $a\in {\mathcal{A}}_{t|t-1}$, the global hypothesis uses the local track hypothesis indicated by ${a}^{i}$. From each track, a single trajectory hypothesis is collected into a global hypothesis, each track has a single trajectory hypothesis of ${h}_{{n}_{t|t-1}}^{i}$. We will show below how the predicted and updated PMBM density is used to track the all trajectories’ set.

#### Prediction steps

The Poisson components at time $t$ can be predicted by combining Eqs. (18) and (34),

39 $$\begin{array}{c}{D}_{t|t-1}^{u}\left(\mathsf{X},\xi \right)={D}_{t}^{b}\left(\mathsf{X},{\xi }_{t}\right)+{\sum }_{{\xi }_{t-1}}\langle {f}_{t|t-1}\left(x|\cdot \right){\eta }_{t|t-1}\left({\xi }_{t}|{\xi }_{t-1}\right)\times {p}_{S,t}\left(\cdot ,{\xi }_{t-1}\right),{D}_{t-1}\left({\cdot ,\xi }_{t-1}\right)\rangle \end{array}$$

The predicted Bernoulli component’s hypothesis weight at time $t$ is equal to that at time $t-1$, therefore, ${\omega }_{t|t-1}^{i,{a}^{i}}={\omega }_{t-1}^{i,{a}^{i}}$. Additionally, the following are the predicted probability of existence and PDF augmented with model, which correspond to Eqs. (19) and (20):

40 $$\begin{array}{c}{r}_{t|t-1}^{i,{a}^{i}}={r}_{t-1}^{i,{a}^{i}}{\sum }_{{\xi }_{t-1}}\langle {P}_{t-1}^{i,{a}^{i}}\left(\cdot ,{\xi }_{t-1}\right),{p}_{S,t}\left(\cdot ,{\xi }_{t-1}\right)\rangle \end{array}$$

41 $$\begin{array}{c}{P}_{t|t-1}^{i,{a}^{i}}\left(\mathsf{X},\xi \right)\propto {\sum }_{{\xi }_{t-1}}\langle {f}_{t|t-1}\left(\mathsf{X}|\cdot \right){\eta }_{t|t-1}\left({\xi }_{t}|{\xi }_{t-1}\right)\times {p}_{S,t}\left({\cdot ,\xi }_{t-1}\right),{P}_{t-1}^{i,{a}^{i}}\left({\cdot ,\xi }_{t-1}\right)\rangle \end{array}$$

$${n}_{{n}_{t|t-1}}^{i}= {n}_{{n}_{t-1|t-1}}^{i},$$

$${h}_{{n}_{t|t-1}}^{i}= {h}_{{n}_{t-1|t-1}}^{i}$$

$${\omega }_{t|t-1}^{i,{a}^{i}}= {\omega }_{t-1|t-1}^{i,{a}^{i}} \forall {a}^{i}$$

#### Update steps

Let $j$ $\in {\mathbb{M}}_{t}=\left\{1,\dots ,{m}_{t}\right\}$ be an index to each measurement and ${m}_{t}$ be number of measurements received at time $t$. Let ${\mathcal{M}}^{t}$ represent a set of tuples $\left(\mathcal{t},j\right)$, where $\mathcal{t}\le t$ and $j\in {\mathbb{M}}_{\mathcal{t}}$. The history of measurements associated with track $i$ in hypothesis ${a}^{i}$ is denoted by ${\mathcal{M}}^{t}\left(i,{a}^{i}\right)\subseteq {\mathcal{M}}^{t}$. Comparing it to point object models, the ${\mathcal{M}}^{t}\left(i,{a}^{i}\right)$ could include many elements that correspond to the same time step. The power set of ${z}_{t}$, denoted as ${\mathcal{P}(z}_{t})$, includes all its subsets. Additionally, we define the $p-th$ as a nonempty element in ${\mathcal{P}(z}_{t})$ as ${\mathbf{Z}}_{t}^{p}$($p\in \{1,\dots ,|{\mathcal{P}(z}_{t})|-1\})$ by arranging the elements of ${\mathcal{P}(z}_{t})$ in a random order; the measurement’s set indices of ${\mathbf{Z}}_{t}^{p}$ is represented as{${j}_{1},{j}_{2},\dots ,{j}_{\left|{\mathbf{Z}}_{t}^{{\varvec{p}}}\right|}\}$. For the undetected objects, the intensity function in Eq. (21) has developed into the subsequent form:

42 $$\begin{array}{c}{D}_{t|t}\left(\mathsf{X},\xi \right)=\left(1-{P}_{D,t}\left(x,{\xi }_{t}\right)\right){D}_{t|t-1}\left(\mathsf{X},{\xi }_{t}\right)\end{array}$$

In the case of misdetection, the update of missed detection hypotheses, $i\in \{1,\dots ,{n}_{t|t-1}\}$,${a}^{i}\in \{1,\dots ,{h}_{{n}_{t|t-1}}^{i}\}$

43 $$\begin{array}{c}{\mathcal{M}}^{t}\left(i,{a}^{i}\right)={\mathcal{M}}^{t-1}\left(i,{a}^{i}\right)\end{array}$$

44 $$\begin{array}{c}{\omega }_{t|t}^{i,{a}^{i},0}={\omega }_{t|t-1}^{i,{a}^{i}}{\rho }_{t}^{i,{a}^{i},0}\end{array}$$

45 $$\begin{array}{c}{r}_{t|t}^{i,{a}^{i},0}={r}_{t|t-1}^{i,{a}^{i}}\zeta /{\rho }_{t}^{i,{a}^{i},0}\end{array}$$

46 $$\begin{array}{c}{P}_{t|t}^{i,{a}^{i},0}\left(\mathsf{X},\xi \right)=\frac{\left(1-{p}_{D,t}\left(\mathsf{X},{\xi }_{t}\right)\right){P}_{t|t-1}^{i,{a}^{i}}\left(\mathsf{X},{\xi }_{t}\right)}{{\rho }_{t}^{i,{a}^{i},0}}\end{array}$$

47 $$\begin{array}{c}{\rho }_{t}^{i,{a}^{i},0}=1-{r}_{t|t-1}^{i,{a}^{i}}+{r}_{t|t-1}^{i,{a}^{i}} \zeta \end{array}$$

48 $$\begin{array}{c}\zeta ={\sum }_{{\xi }_{t-1}}\langle \left(1-{p}_{D,t}\left(\cdot ,{\xi }_{t-1}\right)\right),{P}_{t|t-1}^{i,{a}^{i}}\left(\cdot ,{\xi }_{t-1}\right)\rangle \end{array}$$

For legacy Bernoulli components, $z$ is the measurement that has its existence probability ${r}_{t|t}^{i,{a}^{i}}=1$, which indicates that $i\in \{1,\dots ,{n}_{t|t-1}\}$, ${\widehat{a}}^{i}\in \{1,\dots ,{h}_{t|t-1}^{i}\}$ ,and $p\in \{1,\dots ,|{\mathcal{P}(z}_{t})|-1\}$, where ${\widehat{a}}^{i}$ represents the updated prior hypothesis using the nonempty measurement set ${\mathbf{Z}}_{t}^{p}$,

49 $$\begin{array}{c}{a}^{i}= {\widehat{a}}^{i}+{h}_{t|t-1}^{i} p\end{array}$$

50 $$\begin{array}{c}{\mathcal{M}}^{t}\left(i,{a}^{i}\right)=\left\{\left(t,{j}_{1}\right),\dots ,\left(t,{j}_{\left|{\mathbf{Z}}_{t}^{{\varvec{p}}}\right|}\right)\right\}\bigcup {\mathcal{M}}^{t-1}\left(i,{\widehat{a}}^{i}\right)\end{array}$$

51 $$\begin{array}{c}{\omega }_{t|t}^{i,{a}^{i}}={\omega }_{t|t-1}^{i,{a}^{i}}{r}_{t|t-1}^{i,{\widehat{a}}^{i}}{\sum }_{\xi }\langle \varrho \left(\cdot ,{\xi }_{t}|z\right),1\rangle \end{array}$$

$${P}_{t|t}^{i,{a}^{i}}\left(\mathsf{X},\xi |z\right)\propto \varrho \left(\mathsf{X},{\xi }_{t}|z\right)$$

52 $$\begin{array}{c}\varrho \left(\mathsf{X},{\xi }_{t}|z\right)={p}_{D,t}\left(\mathsf{X},{\xi }_{t}\right){l}_{t}\left(z|\mathsf{X}\right){p}_{t|t-1}^{i,{\widehat{a}}^{i}}\left(\mathsf{X},{\xi }_{t}\right)\end{array}$$

For the new tracks generated by Poisson components, $i\in \{p+{n}_{t|t-1}\}$, $p\in \{1,\dots ,|{\mathcal{P}(z}_{t})|-1\}$, where the new track begins on the measurement set ${({\mathbf{Z}}_{t}^{p})}^{2}$,

$${h}_{t|t}^{i}=2, {\mathcal{M}}^{t}\left(i,1\right)=\phi , {\omega }_{t|t}^{i,1}=1, {r}_{t|t}^{i,1}=0$$

53 $$\begin{array}{c}{\mathcal{M}}^{t}\left(i,2\right)=\left\{\left(t,{j}_{1}\right),\dots ,\left(t,{j}_{\left|{\mathbf{Z}}_{t}^{{\varvec{p}}}\right|}\right)\right\}\end{array}$$

for $${\mathbf{Z}}_{t}^{p}$$=1;

54 $$\begin{array}{c}{r}_{t|t}^{p}\left(z\right)={\mathcal{o}}_{t}\left(z\right)/{\rho }_{t}^{p}\left(z\right)\end{array}$$

55 $$\begin{array}{c}{P}_{t|t}^{P}\left(\mathsf{X},\xi |z\right)=\frac{{p}_{D,t}\left(\mathsf{X},{\xi }_{t}\right) {l}_{t}\left(z|\mathsf{X}\right) {D}_{t|t-1}\left(\mathsf{X},{\xi }_{t}\right)}{{\mathcal{o}}_{t}\left(z\right)}\end{array}$$

56 $$\begin{array}{c}{\rho }_{t}^{P}\left(z\right)={\mathcal{o}}_{t}\left(z\right)+{c}_{t}\left(z\right)\end{array}$$

57 $$\begin{array}{c}{\mathcal{o}}_{t}\left(z\right)={\sum }_{{\xi }_{t-1}}\langle {l}_{t}\left(z|\cdot \right) {p}_{D,t}\left(\cdot ,{\xi }_{t-1}\right), {D}_{t|t-1}\left(\mathsf{X},{\xi }_{t-1}\right)\rangle \end{array}$$

### Discussion

The cost matrix represents the input for Murty’s method, constructed from the probability of each element’s existence inside a defined global hypothesis set. Where the process of generating the cost matrix is identical to that of the standard PMBM filter; however, the parameters in the matrix are calculated by summing all motion models. It’s clear that compared to the standard PMBM filter, the proposed filter is heavier due to the motion model interaction process. Further, gating technology^54^ can eliminate some measurements prior to the update process, which decreases the computational cost by reducing the cost matrix’s dimension. The global hypothesis represents the division of all received measurements into subsets, with each subset hypothesized to connect to a specific potential object. Each measurement that has been received thus far and is hypothesized to belong to the same potential object is explained by each trajectory hypothesis. The weight of global hypothesis $a$ is ${\omega }_{t|t-1}^{a}\propto {\prod }_{i\in {\text{T}}_{t|t-1}}{\omega }_{t|t-1}^{i,{a}^{i}}$, where the weight of the single trajectory hypothesis ${a}^{i}$ from the track $a$ is represented by ${\omega }_{t|t-1}^{i,{a}^{i}}$. The single trajectory density and the Poisson RFS’s intensity combine to generate a mixed density of the form

58 $$\begin{array}{c}f\left(\mathsf{X}\right)= \sum_{t}{\omega }^{t}{f}^{t}\left({x}_{\beta :\epsilon }|\beta ,\epsilon \right){\Delta }_{{e}^{t}}\left(\epsilon \right){\Delta }_{{b}^{t}}\left(\beta \right)\end{array}$$

where each component of the mixture is identified by its weight ${\omega }^{t}$, a distinct time of birth ${b}^{t}$, a distinct time of most recent ${e}^{t}$ where ${b}^{t}\le {e}^{t}$ for all $t$, and a density of state sequences ${f}^{t}(\cdot )$.For the weights $\sum_{t}{\omega }^{t}$ we have:

- $\sum_{t}{\omega }^{t}=1,$ if $f\left(\cdot \right)$ is a density.
- $\sum_{t}{\omega }^{t}\ge 0,$ if $f\left(\cdot \right)$ is an intensity.

where the state sequence ${x}_{\beta :\epsilon }$ can be represented simply with this type of state density, dependent on $\beta$ and $\epsilon$. The implementation of the proposed MM-TPMBM filter can be achieved through the application of a particle-based Sequential Monte Carlo (SMC) approximation, as detailed in reference^55,56^. Within this SMC framework, the model variable is intrinsically associated with the state-weight pairs of each particle. Specifically, each particle represents a potential state of the system, and its corresponding weight reflects the likelihood of that state given the observational data. This establishes a direct link between the model variable and the particle-based representation, allowing the filter to track the system’s evolution by propagating and updating these particles over time in accordance with the underlying dynamics and incoming observations. However, despite its conceptual appeal, this particle-based approach is not without significant limitations. A primary challenge lies in the necessity for a substantial number of particles to ensure that the approximation remains sufficiently accurate, particularly in systems characterized by high-dimensional state spaces. This requirement imposes considerable computational demands, as each particle must be individually processed propagated through the system model and reweighed at every time step.

**Fig. 2:**
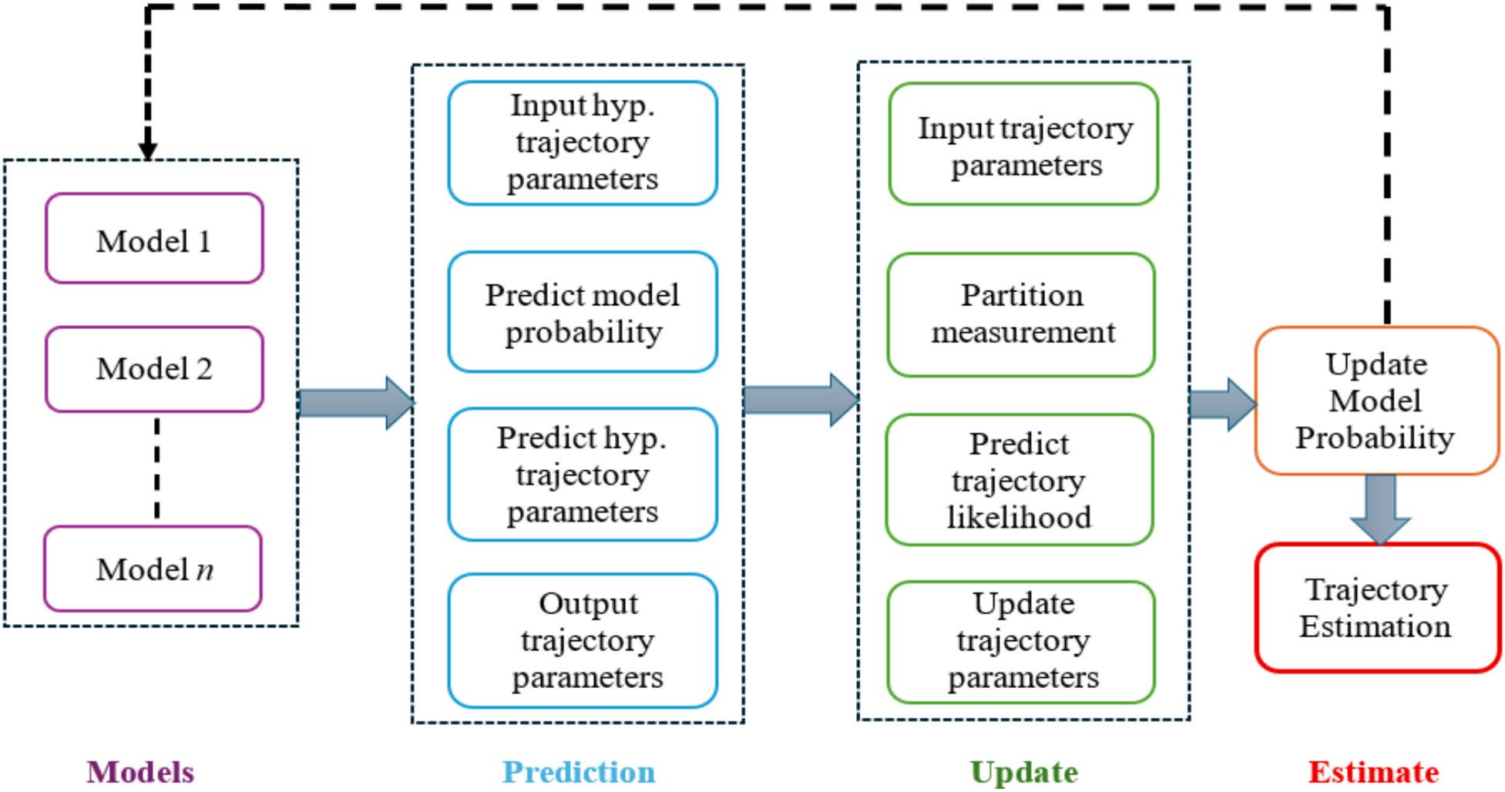
Flowchart of the Multiple-Model Trajectory Poisson Multi-Bernoulli Mixture.

Furthermore, the extraction of state estimates from the particle set typically relies on clustering algorithms, which often yield inconsistent results. The variability in clustering outcomes can be particularly problematic in complex scenarios where the particle distribution exhibits multimodal characteristics or significant dispersion, thereby compromising the reliability of the filter’s output. In contrast, Gaussian Mixture (GM) approximations^57,58^, present a robust alternative that effectively mitigates these challenges. By representing the probability distributions as a finite mixture of Gaussian components, GM approximations can achieve comparable levels of accuracy with a markedly reduced computational footprint relative to particle-based methods. This is because GM approximations require fewer components to model the distribution effectively, thereby alleviating the computational burden associated with large particle sets. Moreover, the process of state extraction is streamlined within the GM framework, as estimates can be derived directly from the mixture model such as by computing the mean of the mixture or identifying the most probable Gaussian component thus circumventing the inconsistencies inherent in clustering-based approaches. Figure 2 illustrates one complete recursive iteration, beginning with the prediction step that uses a set of $n$ motion models to predict trajectory parameters and model probabilities. The subsequent update step incorporates the measurement set to refine the predicted likelihoods. The cycle concludes with updating the model probabilities and extracting the final estimated trajectories.

## The implementation

This section discusses the several techniques that can be utilized to keep the computational complexity of the proposed MM-TPMBM filter at an acceptable level. Additionally, we denoted the implementation of the MM-TPMBM filter that is based on Gaussian technique.

### Gaussian implementation

In the Gaussian implementation, we consider information from all detected trajectories as well as information about undetected trajectories that are still alive. In most real scenarios, undetected and non-existent trajectories are useless, only live trajectories are taken into consideration by the PPP. The augmented state’s transition density is assumed to satisfy:

59 $$\begin{array}{c}{f}_{t|t-1}\left({\mathsf{X}}_{t},{\xi }_{t}|{\mathsf{X}}_{t-1},{\xi }_{t-1}\right)={\eta }_{t|t-1}\left({\xi }_{t}|{\xi }_{t-1}\right) N\left(\mathsf{X};{F}_{t}\left({\xi }_{t}\right) {\mathsf{X}}_{t-1},{\mathcal{Q}}_{t}\left({\xi }_{t}\right)\right)\end{array}$$

where $\mathcal{N}(\cdot )$ is the Gaussian distribution, ${F}_{t}\left(\xi \right)$ represents the transition matrix of the motion model associated with $\xi$, and ${\mathcal{Q}}_{t}(\xi$) denotes the covariance matrix of the process noise related to model $\xi$, it is assumed that the likelihood function has a Gaussian form

60 $$\begin{array}{c}{l}_{t}\left(z|x\right)= N\left(z; {H}_{t}{\mathsf{X}}_{t}, {R}_{t}\right)\end{array}$$

where ${H}_{t}$ is the measurement model, and ${R}_{t}$ is the process noise covariance. Furthermore, it is usually assumed that the survival and detection probabilities depend on the model, that is ${p}_{D,t}\left(\mathsf{X},\xi \right)={p}_{D,t}\left(\xi \right)$ and ${p}_{S,t}\left(\mathsf{X},\xi \right)={p}_{S,t}\left(\xi \right)$. We recommend the reader to^58,59^ for more details about the Gaussian implementation of the PMBM filter.

#### Predict

For the set of all trajectories in the Gaussian implementation, we consider data from all detected trajectories as well as data from undetected alive trajectories. The Gaussian mixture (GM) is employed to denote both the probability hypothesis density of the birth density at time step $t$ and posterior intensity at time $t-1$, given as follows:

61 $$\begin{array}{c}{D}_{t}^{b}\left(\mathsf{X},\xi \right)={\eta }_{t}^{b}\left(\xi \right)\sum_{q=1}^{{n}_{t}^{b}\left(\xi \right)}{\omega }_{t}^{b,q}\left(\xi \right)N\left(\mathsf{X};{\mathsf{X}}_{t}^{b,q}\left(\xi \right),{P}_{t}^{b,q}\left(\xi \right)\right)\end{array}$$

62 $$\begin{array}{c}{D}_{t-1}\left(\mathsf{X},\xi \right)=\sum_{q=1}^{{n}_{t-1}^{p}\left(\xi \right)}{\omega }_{t-1}^{p,q}\left(\xi \right)N\left(\mathsf{X};{\mathsf{X}}_{t-1}^{p,q}\left(\xi \right),{P}_{t-1}^{p,q}\left(\xi \right)\right)\end{array}$$

where the probability of the initial transition of model $\xi$ is represented by ${\eta }_{t}^{b}\left(\xi \right)$. The number of Gaussian components (GCs) for the birth model is denoted by ${n}_{t}^{b}\left(\xi \right)$, while the posterior intensity under model is represented by ${n}_{t-1}^{p}$, and both ${\omega }_{t}^{b,q}$ and ${\omega }_{t-1}^{p,q}$ represent weights of the $q\text{th}$ component, with ${\mathsf{X}}_{t-1}^{q}(\xi )$ and ${P}_{t-1}^{q}(\xi )$ indicating the mean and covariance matrix, respectively. As a result, the predicted intensity is also in a Gaussian form,

63 $$\begin{array}{c}{D}_{t|t-1}\left(\mathsf{X},\xi \right)={D}_{t}^{b}\left(\mathsf{X},\xi \right)+\sum_{{\xi }_{t-1}}\sum_{q=1}^{{n}_{t-1}^{p}\left({\xi }_{t-1}\right)}{\omega }_{t|t-1}^{p,q}\left({\xi }_{t}|{\xi }_{t-1}\right)\times \mathcal{N}\left(\mathsf{X};{\mathsf{X}}_{t|t-1}^{p,q}\left({\xi }_{t}|{\xi }_{t-1}\right),{P}_{t|t-1}^{p,q}\left({\xi }_{t}|{\xi }_{t-1}\right)\right)\end{array}$$

where,

64 $$\begin{array}{c}{\mathsf{X}}_{t|t-1}^{p,q}\left({\xi }_{t}|{\xi }_{t-1}\right)={F}_{t}\left({\xi }_{t}\right) {\mathsf{X}}_{t-1}^{p,q}\left({\xi }_{t-1}\right)\end{array}$$

65 $$\begin{array}{c}{P}_{t|t-1}^{p,q}\left({\xi }_{t}|{\xi }_{t-1}\right)={\mathcal{Q}}_{t}\left(\xi \right)+{F}_{t}\left({\xi }_{t}\right){P}_{t-1}^{p,q}\left({\xi }_{t-1}\right){F}_{t}{\left({\xi }_{t}\right)}^{\text{T}}\end{array}$$

66 $$\begin{array}{c}{\omega }_{t|t-1}^{p,q}\left({\xi }_{t}|{\xi }_{t-1}\right)={p}_{S,t}\left({\xi }_{t-1}\right){\eta }_{t|t-1}\left({\xi }_{t}|{\xi }_{t-1}\right){\omega }_{t-1}^{p,q}\left({\xi }_{t-1}\right)\end{array}$$

For the Bernoulli component, the predicted probability of existence and the density function are satisfied as follows:

67 $$\begin{array}{c}{r}_{t|t-1}^{i,{a}^{i}}={r}_{t-1}^{i,{a}^{i}}\sum_{{\xi }_{t-1}}\sum_{q=1}^{{n}_{t-1}^{i,{a}^{i}}\left({\xi }_{t-1}\right)}{\omega }_{t|t-1}^{i,{a}^{i},q}\left({\xi }_{t-1}\right) {p}_{S,t}\left({\xi }_{t-1}\right)\end{array}$$

68 $$\begin{array}{c}{P}_{t|t-1}^{i,{a}^{i}}\left(\mathsf{X},\xi \right)=\sum_{{\xi }_{t-1}}\sum_{q=1}^{{n}_{t-1}^{i,{a}^{i}}\left({\xi }_{t-1}\right)}{\omega }_{t|t-1}^{i,{a}^{i},q}\left({\xi }_{t}|{\xi }_{t-1}\right)\times \mathcal{N}\left(\mathsf{X};{\mathsf{X}}_{t|t-1}^{i,{a}^{i},q}\left({\xi }_{t}|{\xi }_{t-1}\right),{P}_{t|t-1}^{i,{a}^{i},q}\left({\xi }_{t}|{\xi }_{t-1}\right)\right)\end{array}$$

Moreover, the predicted Bernoulli component’s hypothesis weight remains constant, that is ${\omega }_{t|t-1}^{i,{a}^{i}}={\omega }_{t-1}^{i,{a}^{i}}$. It is important to note that^59^ the hypothesis weight and existence probability of Bernoulli components are not dependent on model $\xi .$

#### Update

We reformulate the predicted intensity as,

69 $$\begin{array}{c}{D}_{t|t-1}\left(\mathsf{X},\xi \right)=\sum_{q=1}^{{n}_{t|t-1}^{p}\left(\xi \right)}{\omega }_{t|t-1}^{p,q}\left(\xi \right)N\left(\mathsf{X};{\mathsf{X}}_{t|t-1}^{p,q}\left(\xi \right),{P}_{t|t-1}^{p,q}\left(\xi \right)\right)\end{array}$$

Thus, the updated posterior intensity ${D}_{t}\left(\mathsf{X},\xi \right)$ is also a Gaussian mixture, as shown by:

70 $$\begin{array}{c}{D}_{t}\left(\mathsf{X},\xi \right)=\sum_{q=1}^{{n}_{t}^{p}\left(\xi \right)}{\omega }_{t}^{p,q}\left(\xi \right)N\left(\mathsf{X};{\mathsf{X}}_{t}^{p,q}\left(\xi \right),{P}_{t}^{p,q}\left(\xi \right)\right)\end{array}$$

where,$${n}_{t}^{p}\left(\xi \right)={n}_{t|t-1}^{p}\left(\xi \right), {\mathsf{X}}_{t}^{p,q}\left(\xi \right)={\mathsf{X}}_{t|t-1}^{p,q}\left(\xi \right), {P}_{t}^{p,q}\left(\xi \right)={P}_{t|t-1}^{p,q}\left(\xi \right)$$

71 $$\begin{array}{c}{\omega }_{t}^{p,q}\left(\xi \right)={\omega }_{t|t-1}^{p,q}\left(\xi \right) \left(1-{p}_{D,t}\left({\xi }_{t}\right)\right)\end{array}$$

For the remaining updated Bernoulli components, we employ the same method as discussed in Eqs. (43–57) in Gaussian form.

### Practical data association considerations

The practical issues that need to be considered to improve the computational efficiency of the TPMBM filter are discussed in this section. If the trajectory lengths continue to grow and the covariance matrix sizes continue to increase quadratically, then the computation of the filtering recursion will become increasingly computationally expensive over time. This will result in a reduction in the total number of global hypotheses because it is only possible to consider data association events that have a high likelihood.

As can be observed in^50,60^, the data association problem is often tackled in two stages with distinct phases. First, clustering algorithms^61^ are utilized to identify several configurations for clustering the measurements. Second, assignment methods, such as Murty’s algorithm^53^, which assigns measurement clusters to objects according to the assumption that a track can be assigned to a single measurement cluster. This method is used to sequentially match the generated global hypothesis by the filtering process in an efficient manner. Because of this, we use Murty’s method to select the most appropriate new global hypothesis, without considering the relative importance of each new global hypothesis. It is possible to use random sampling methods^62^ as an alternative to clustering and assignment to identify a subset of associations. This is because these methods directly optimize the likelihood of the data being associated with associations. As a consequence of this, it is feasible to efficiently and accurately track the objects and to obtain the greatest possible correspondence link between the measurements and the objects.

The quantity of Gaussian components (GCs) in the MM-TPMBM filter, relevant to both Poisson and Bernoulli components, is dependent upon the motion model. In particular, the MM-TPMBM filter includes Gaussian mixtures for all motion models, compared to the standard PMBM filter, which treats each single-object hypothesis just as a linear combination of Gaussian components. Consequently, the MM-PMBM filter in the GM implementation has a higher computational cost than the conventional PMBM filter. An updated PMBM density estimate causes the number of parameters (GCs) to grow exponentially with time, resulting in an increased computational cost in subsequent steps. The global hypotheses with updated weights that are lower than a given threshold is pruned^53,57^. The symmetry of the posterior is not affected by the pruning process. Single trajectory hypotheses that do not belong to the remaining global hypotheses can therefore be pruned using this method. More than that, we eliminate Bernoulli components that have a probability of existence that is lower than a particular threshold. The mixture representation of the Poisson RFS intensity is pruned to remove the components that have weights that are lower than a certain threshold value. Because the aim of merging is to maintain some information from each merged component, we finally merge components that have a weight that is comparable to each other, or we merge the approximate sums of components into a single component^30,57^.

### Pseudocode for the gaussian MM-TPMBM filter

By using the track-oriented (TO) object PMBM trackers, a track is initiated at time $t$ for each non-empty subset of the measurement set ${{\varvec{Z}}}_{t}$. A look-up table is used to represent global hypothesis in the TO implementation. The $\left(a,i\right)th$ entry of the look-up table denotes the index of the single trajectory hypothesis within the $i th$ track that is included in the $a th$ global hypothesis. If the $a th$ global hypothesis excludes a single trajectory hypothesis from the $i th$ track, then the $(a,i) th$ entry of the look-up table will be zero. The single existence hypothesis may be pruned due to its low existence probability, or it may be the case of a non-existent single trajectory hypothesis. The pseudocode for the Gaussian MM-TPMBM filter is outlined in Algorithm 1.

**Algorithm 1:**
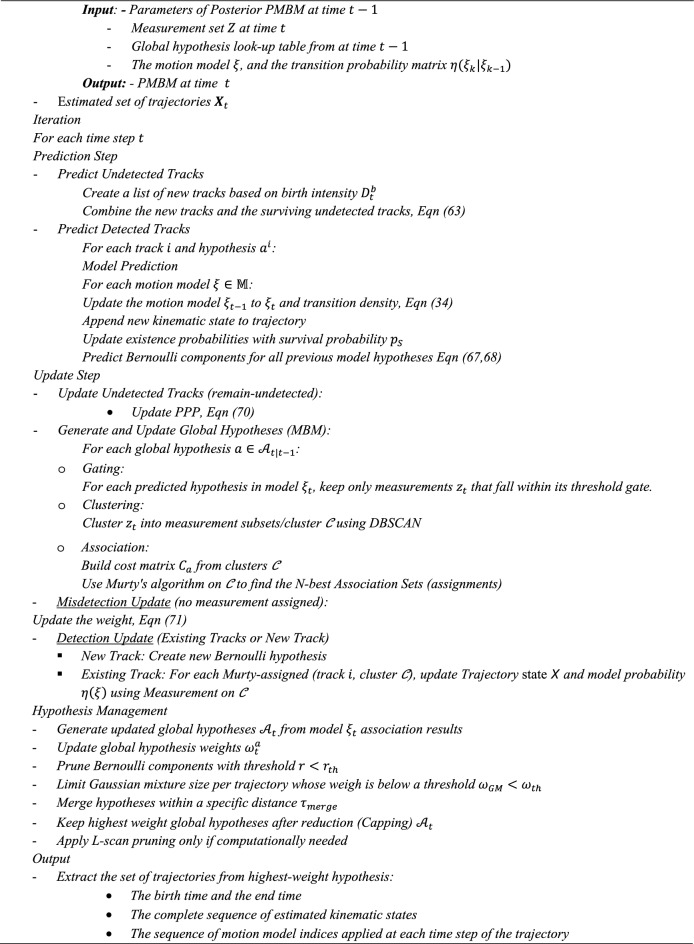
Gaussian MM-TPMBM Filter Pseudocode.

## Simulation and results

In this section, we report the outcomes of comprehensive Monte Carlo simulations conducted to benchmark the tracking performance of four distinct multi‐object filters under identical experimental conditions. We compare:

- The proposed MM‑TPMBM filter, which integrates multiple motion models within the trajectory PMBM framework.
- The MM‑PMBM filter^59^, which similarly employs multiple models but without explicit trajectory estimation.
- The TPMBM filter^27^, i.e. the standard PMBM filter applied to all trajectories using a one‐scan Gaussian approximation of the state‐sequence density (*L* = 1).
- The δ‑GLMB filter^44,45^, which models the multi‐object state as a labeled RFS and performs joint prediction and update.

The δ‑GLMB implementation generates data‐association hypotheses via Murty’s ranked assignment algorithm, retaining the top 1000 global hypotheses to balance performance and computational tractability. For track extraction, we employ a maximum a posteriori (MAP) estimator of the object cardinality^27^: first determining the most probable number of objects, then selecting the highest‐weight hypothesis corresponding to that cardinality. Subsequent sections detail the simulation parameters such as clutter rates, detection probabilities, and motion model transition rates and present comparative results in terms of localization error, cardinality estimation accuracy, root‐mean‐square GOSPA error, and execution time.

### Simulation scenario

**Fig. 3:**
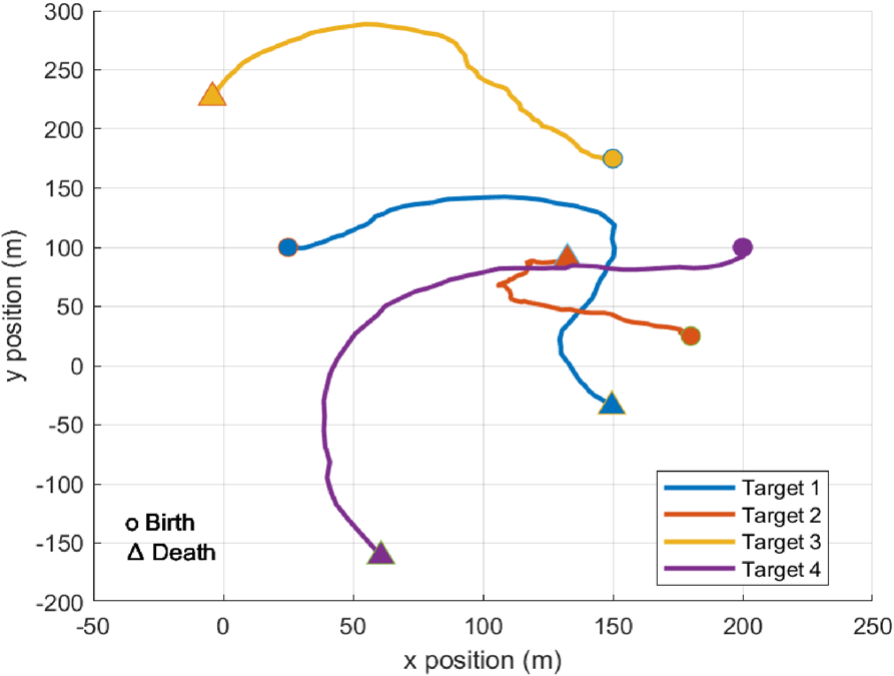
Four target trajectories in *x-y* plane, start/end denoted by 0/∆ (Scenario 1).

As shown in Fig. 3, we have a two-dimensional scenario space with dimensions of $250m$ each, and four objects are moving around the observation area. The state of each object has a two-dimensional location and velocity, i.e., $x={\left[{p}_{x},{\dot{p}}_{x},{p}_{y},{\dot{p}}_{y}\right]}^{\text{T}}$, the motion model $\xi$ determines how the objects move, i.e.,

72 $$\begin{array}{c}{x}_{t}={\eta }_{t}\left(\xi \right){x}_{t-1}+{u}_{t}\end{array}$$

where ${u}_{t}\sim \mathcal{N}(0,{\sigma }_{v}^{2}{I}_{2})$, ${\sigma }_{v}=2m$ and ${I}_{n}$ denote the $n\times n$ identity matrices, the observed time is T_0_=81s.

$$H = \left[ {\begin{array}{*{20}c} 1 & 0 & 0 & 0 \\ 0 & 0 & 1 & 0 \\ \end{array} } \right],\quad R = \sigma_{\varepsilon } \left[ {\begin{array}{*{20}c} 1 & 0 \\ 0 & 1 \\ \end{array} } \right]$$

where $H$ represents observation matrix, and $R$ is the measurement noise covariance matrix, and ${\sigma }_{\varepsilon }=10m$ stands for the measurement noise standard deviation. The survival probability for is ${p}_{S}=0.99$ and and the probability of detection is ${p}_{D}=0.9$, and Poisson clutter is distributed uniformly with rate ${\lambda }_{C}=10$.

The δ-GLMB filter needs a (labelled) multi-Bernoulli model instead of a Poisson birth model. In the domain of multi-object tracking, the maneuvering dynamics of each object are characterized by stochastic transitions among three distinct motion models. Specifically, the first model is designated as a constant velocity (CV) model, which presupposes a linear trajectory with an unvarying speed. The second model is identified as a constant turn (CT) model, distinguished by a clockwise turn rate θ$=4 deg/sec$ that facilitates circular motion in a specified direction. The third model, termed the counter constant turn (CCT) model, is characterized by a counterclockwise turn rate quantified at $\uptheta =-4 deg/sec$, thereby enabling circular motion in the opposing direction.

The mathematical underpinnings of these models, encompassing the state transition matrices and the process noise covariance matrices pertinent to the CV and CT models, are thoroughly detailed as follows:

$${F}_{CV}=\left[\begin{array}{cccc}1& T& 0& 0\\ 0& 1& 0& 0\\ 0& 0& 1& T\\ 0& 0& 0& 1\end{array}\right],{F}_{CT}=\left[\begin{array}{cccc}1& (\text{sin}\theta T)/\theta & 0& -(1-\text{cos}\theta T)/\theta \\ 0& \text{cos}\theta T& 0& -\text{sin}\theta T\\ 0& (1-\text{cos}\theta T)/\theta & 1& (\text{sin}\theta T)/\theta \\ 0& \text{sin}\theta T& 0& \text{cos}\theta T\end{array}\right]$$

$$Q_{CV} = \sigma_{CV}^{2} \left[ {\begin{array}{*{20}c} {T^{4} /4} & {T^{3} /2} & 0 & 0 \\ {T^{3} /2} & {T^{2} } & 0 & 0 \\ 0 & 0 & {T^{4} /4} & {T^{3} /2} \\ 0 & 0 & {T^{3} /2} & {T^{3} /2} \\ \end{array} } \right],\quad Q_{CT} = \sigma_{CT}^{2} \left[ {\begin{array}{*{20}c} {T^{4} /4} & {T^{3} /2} & 0 & 0 \\ {T^{3} /2} & {T^{2} } & 0 & 0 \\ 0 & 0 & {T^{4} /4} & {T^{3} /2} \\ 0 & 0 & {T^{3} /2} & {T^{3} /2} \\ \end{array} } \right]$$

where $T=1s$ indicates the sample rate. The Poisson birth intensity constitutes a Gaussian mixture,

$${D}_{t}^{b}={\sum }_{i=1}^{4}{\mathcal{w}}_{b} \mathcal{N}(x;{m}_{b}^{\left(i\right)},{P}_{b})$$

where _b_=0.1 is the probability of birth**,** and ${P}_{b}=\text{diag}([150, 1, 150, 1{]}^{\text{T}}{)}^{2}$ represents the covariance. The distribution of the three models at birth is

$${\eta }_{t}^{b}\left(\xi \right)=\left[\begin{array}{ccc}0.45& 0.35& 0.35\end{array}\right]$$

Furthermore, the initial locations and the survival time for four objects and their kinematic states are described as follows:

- First object is born at 1 s; its initial location is $$[\begin{array}{cccc}25& 1.25& 100& 0\end{array}{]}^{\mathbf{T}}$$, and it dies at 60 s.
- Second object is born at 10 s; its initial location is $$[\begin{array}{cccc}180& 0& 25& 0.125\end{array}{]}^{\mathbf{T}}$$, and it dies at 70 s.
- Third object is born at 20 s; its initial location is $$[\begin{array}{cccc}150& -1.25& 175& 0\end{array}{]}^{\mathbf{T}}$$, and it dies at 81 s.
- Fourth object is born at 30 s; its initial location is $$[\begin{array}{cccc}200& 0& 100& -0.125\end{array}{]}^{\mathbf{T}}$$, and it dies at 81 s.

All objects follow the motion model; follow CV model till the time $t=15s$, then a maneuver happens and the motion model turns to CT model with a clockwise turn rate of $\theta =4 deg/sec$ till the time $t=45s$, finally another happens and the motion model turns to CCT model with a counterclockwise turn rate of $\theta =-4 deg/sec$ till the scenario. The Markovian transition probability matrix $\eta ({\xi }_{t}|{\xi }_{t-1})$ defines the transition between the motion models as follows,

$${\eta }_{t|t-1}\left({\xi }_{t}|{\xi }_{t-1}\right)=\left[\begin{array}{ccc}0.70& 0.15& 0.15\\ 0.15& 0.70& 0.15\\ 0.15& 0.15& 0.70\end{array}\right]$$

### Performance evaluation

**Fig. 4:**
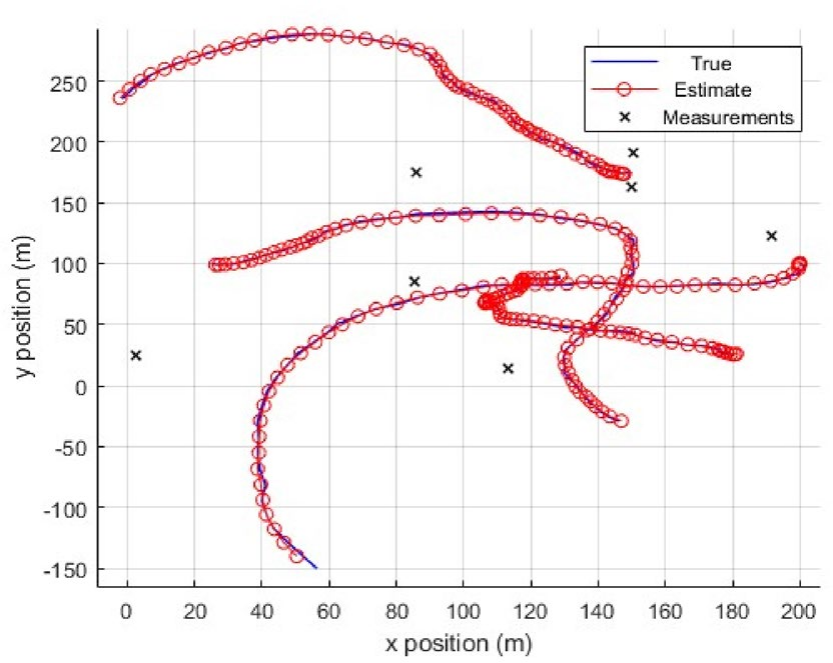
Generated measurement data (red circles).

We evaluate four true trajectories, derived from sampling the dynamic model, as illustrated in Fig. 4. First, we examine a single realisation of the measurements to facilitate the plotting of the posterior means of the trajectories corresponding to the global hypothesis with the highest weight at the conclusion of the simulation. The scenario is challenging for the $\updelta$-GLMB filter due to the large number of global hypotheses included in the initial update step as there is multiple potential *i.i.d* birth with large spatial uncertainty. To run this filter at a reasonable time, potentially relevant information must be pruned, which represents a performance loss. On the other hand, the PMBM and TPMBM updates only need a single global hypothesis at the first time step, which contains all the information and is very fast for computation. We concentrate on comparing tracking performance that takes into consideration all errors between the true set of tracks and the estimated tracks. The introduction of trajectory sets facilitates the utilization of metrics-based definitions for estimation error^63–65^. It can also be utilized with different measurement models, like track-before-detect, or to develop new algorithms, like the TPHD filter, that do not result in an MHT-type framework, in contrast to classical MHT algorithms. The linear programming (LP) metric $d(\cdot ,\cdot )$ for trajectory sets^63^ with parameters $p=2$, $c=20$, and switch cost γ = 1 is the best trajectory metric (TM) used to calculate the error. The switching costs for tracks are minimal and relatively uniform across every filter based on trajectory sets. The δ-GLMB/LMB filter is the only filter that has track switches before objects nearing proximity, due to the *i.i.d* newborn objects, and has the highest switching costs. In our results, we compute $d(\cdot ,\cdot )$ using only the position elements and normalize the error by the time window under consideration, making the squared error at time $t$ equal to ${d}^{2}({{\varvec{X}}}_{t},{\widehat{{\varvec{X}}}}_{t})/k$, where ${{\varvec{X}}}_{t},{\widehat{{\varvec{X}}}}_{t}$ denote the true set and the estimate set, respectively. At a specific time step, the root mean square (RMS) error is

73 $$\begin{array}{c}d\left(t\right)=\sqrt{\frac{1}{{N}_{mc}t} \sum_{i=1}^{{N}_{mc}}{d}^{2}\left({{\varvec{X}}}_{t},{\widehat{{\varvec{X}}}}_{t}^{i}\right)}\end{array}$$

where ${N}_{mc}$ is the number of Monte Carlo simulation runs (100), ${\widehat{{\varvec{X}}}}_{t}^{i}$ is the estimate of the trajectory set at time $t$ in the $i-th$ Monte Carlo run. The RMS error considering all time steps is

74 $$\begin{array}{c}{d}_{T}=\sqrt{\frac{1}{{N}_{S}} \sum_{t=1}^{{N}_{S}}{d}^{2}\left(t\right)}\end{array}$$

where ${d}_{T}$ is the trajectory error used for all trajectories, and ${N}_{S}$ is the time steps $(81s)$.

In addition to the LP metric, we analyze the error in the final trajectory estimates derived from the trajectory filters utilizing Monte Carlo simulation with ${N}_{mc}=100$ runs. To accomplish this, we calculate the root mean square generalized OSPA error^65^. The Generalized Optimal Sub Pattern Assignment (GOSPA) metric employs parameters $p=2$, $c=20$ and $\alpha =2$, as this selection facilitates the decomposition of the average GOSPA error, missed object error (MTE), localisation error (LE), and false object error (FTE) and cardinality estimate. The RMS GOSPA error across all time steps is

75 $$\begin{array}{c}RMSGOSPA=\sqrt{\frac{1}{{N}_{mc}t} \sum_{i=1}^{{N}_{mc}}\sum_{t=1}^{t}{d}^{2}\left({{\varvec{X}}}_{t},{\widehat{{\varvec{X}}}}_{t}^{i}\right)}\end{array}$$

### MM-TPMBM evaluation

**Fig. 5:**
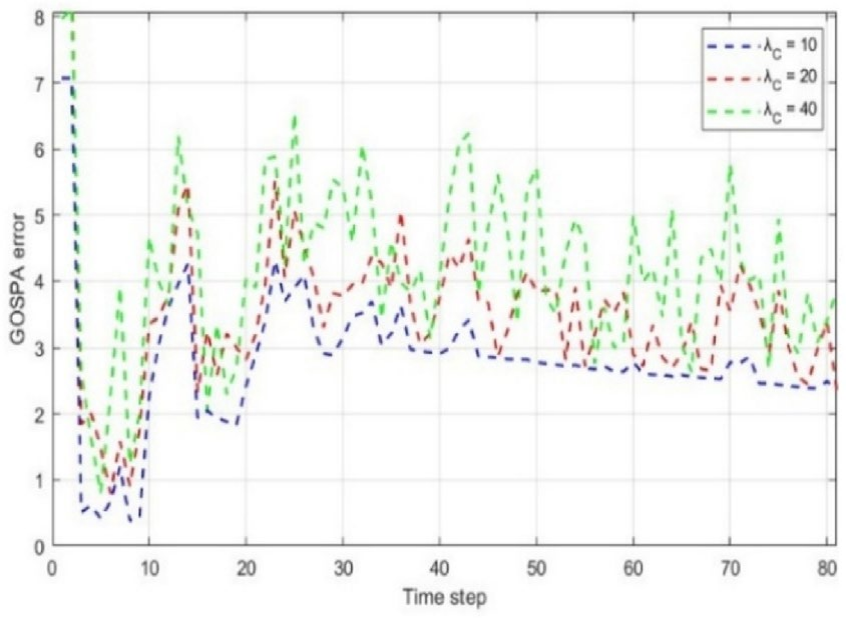
GOSPA error for MM-TPMBM filter at different clutter rates.

The performance of the proposed MM-TPMBM filter under varying clutter rates is demonstrated in Fig. 5. At the clutter rate ${\lambda }_{C}=10$, the filter exhibits stable behavior, with the GOSPA error converging to an average of approximately 2.5–3.0 after the initial transient phase; in this case, nearly 75% of the error values remain below 3, indicating high localization accuracy in relatively clean environments. When the clutter rate is increased to ${\lambda }_{C}=20$, a slight degradation is observed, with the average error rising to approximately 3.0–3.5, while about 65% of the error values remain below 3.5. This demonstrates that the filter preserves satisfactory tracking performance under moderate clutter. By contrast, at a high clutter rate ${\lambda }_{C}=40$, the average error increases substantially to approximately 4.5–5.0, with frequent peaks exceeding 6 and fewer than 30% of the error values remaining below 3.5. These findings demonstrate that while the proposed filter achieves reliable performance at low and medium clutter levels, its accuracy diminishes considerably under highly cluttered conditions.

**Fig. 6:**
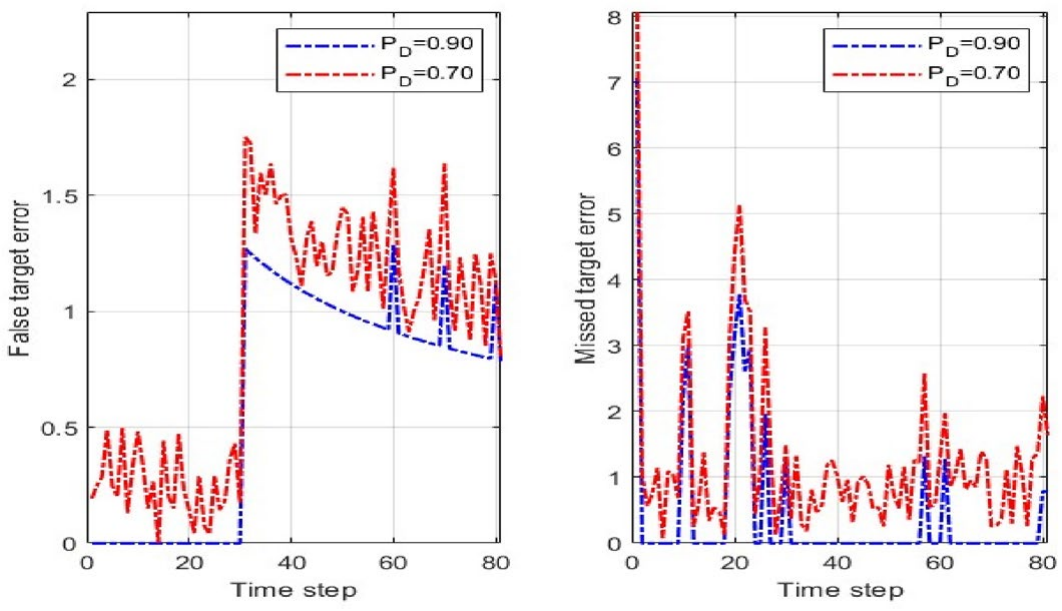
FTE and MTE for MM-TPMBM filter at probability of detection.

Figure 6 illustrates the performance of the proposed MM-TPMBM filter under different probabilities of detection. It reveals distinct impacts of detection probabilities ${p}_{D}$ on false and missed target errors. For false target error, ${p}_{D}=0.90$ produces an initial peak of 1.5 at time step 28, declining to an average of 0.45 with fluctuations below 0.3, whereas ${p}_{D}=0.70$ exhibits a 33% higher peak (2.0) and a 22% greater average error (0.55), indicating increased early-phase spurious detection persistence. Conversely, missed target error shows a more pronounced effect: ${p}_{D}=0.90$ maintains an average of 1.6 with a peak of 4.0, while ${p}_{D}=0.70$ escalates to an average of 3.1 which reflects a 70% increase in error due to the lower detection probability. These results confirm that reducing the detection probability significantly degrades tracking accuracy by increasing both false and missed target errors.

**Fig. 7:**
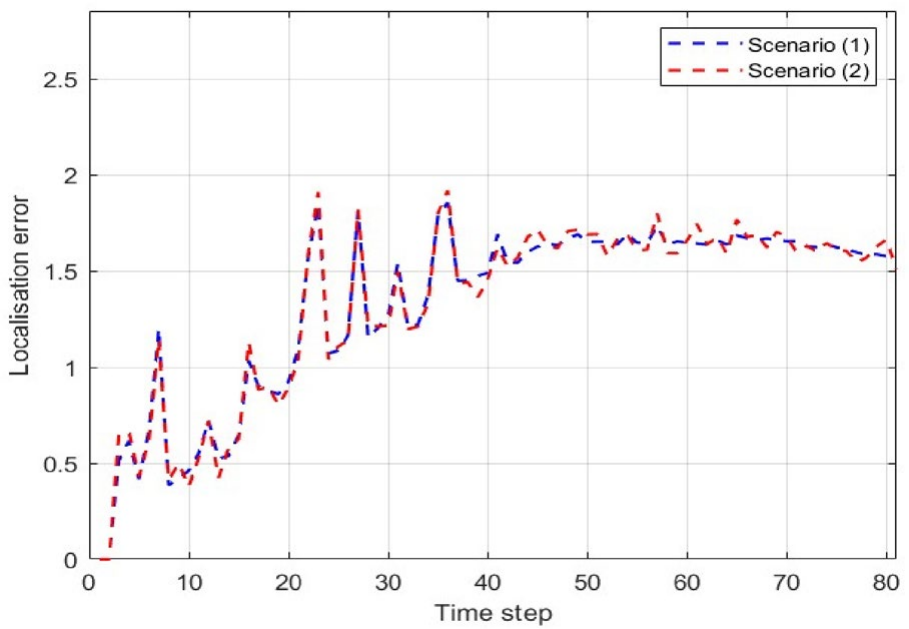
LE for MM-TPMBM filter at different two scenarios.

**Fig. 8:**
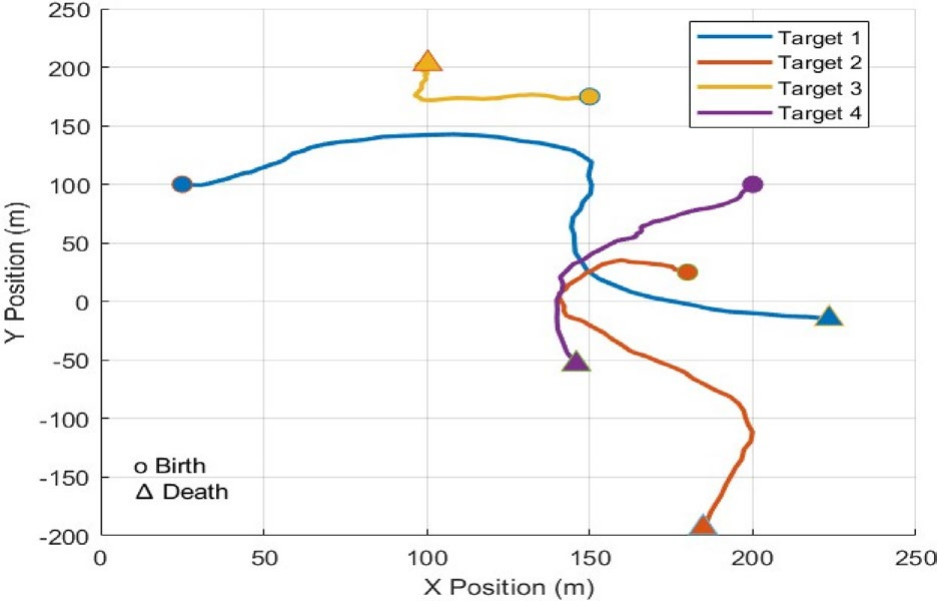
Four target trajectories in *x-y* plane (Scenario 2).

Figure 7 shows the localisation error (LE) of the proposed MM-TPMBM filter across two distinct scenarios. The results indicate that the filter’s performance is robust and largely unaffected by the differences between the two scenarios. In Scenario 1, LE generally fluctuates between approximately 0.25 and 1.75, with a mean value of around 1.2. Similarly, in Scenario 2, shown in Fig. 8, the LE exhibits a comparable trend, with its values ranging from 0.25 to 1.9. After the initial transients, both scenarios converge to a steady-state error of approximately 1.6 after time step 40. The average difference between the two scenarios is negligible, approximately 1.5% over the full simulation. This minimal deviation confirms that the proposed filter maintains a consistent and stable level of performance, regardless of the specific conditions presented by the two scenarios.

### Comparison analysis

A comprehensive comparison analysis performance evaluation was conducted to assess the effectiveness of the proposed MM-TPMBM filter in comparison with three established multi-object tracking filters: TPMBM, MM-PMBM, and δ-GLMB. All filters were tested under identical experimental conditions involving four maneuvering objects with varying birth and death times.

**Fig. 9:**
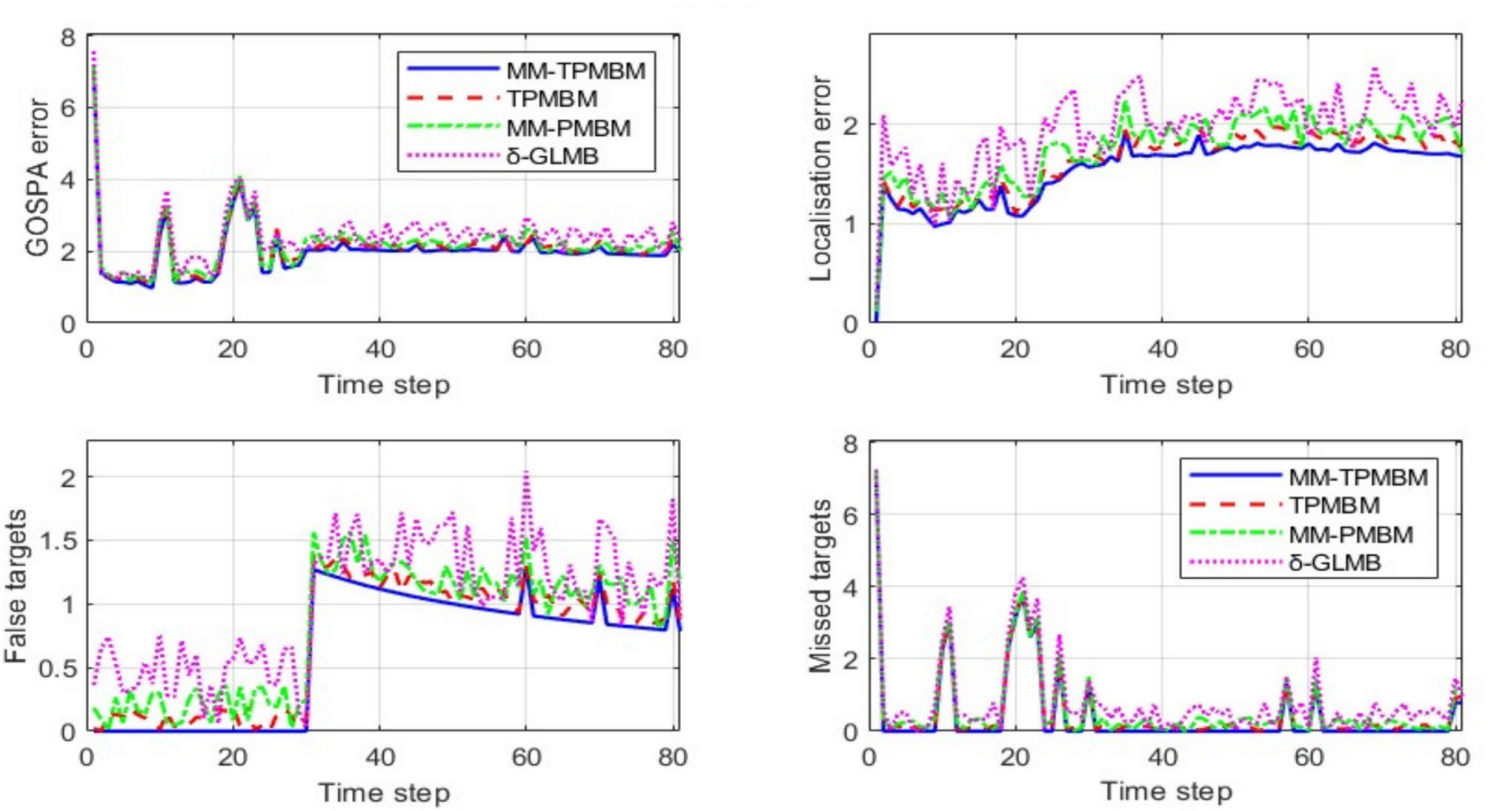
Comparisons of GOSPA errors, including total error, localization error, false target error, and missed target error against time based on 100 Monte Carlo runs.

Figure 9 illustrates that the MM-TPMBM filter outperforms the other filters across all evaluated metrics, where the MM-TPMBM filter records the lowest mean Total GOSPA Error at 2.263, surpassing TPMBM (2.489, 9% higher), MM-PMBM (2.718, 17% higher), and δ-GLMB (3.021, 25% higher). The MM-TPMBM filter outperforms the other filters, boasting a mean LE of 1.5347, 9% better than TPMBM’s 1.688, 17% better than MM-PMBM’s 1.845, and 27% better than δ-GLMB’s 2.098 demonstrating its remarkable precision, especially during the different objects’ births and deaths, such as the congestion at time step 30 with all four objects present.

**Fig. 10:**
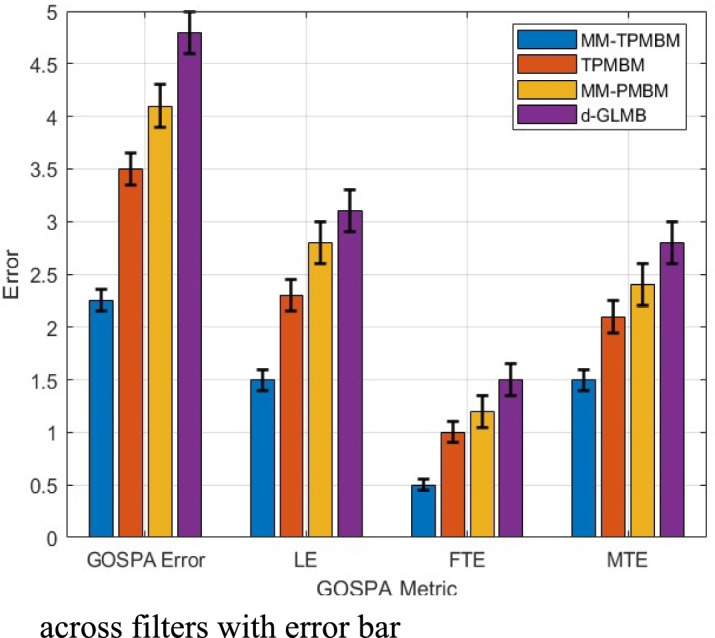
Comparison of GOSPA error metrics across filters with error bar.

Moreover, the MM-TPMBM filter offers the minimum FTE, achieving a mean of 0.56578 compared to 0.622 (TPMBM, 9% higher), 0.679 (MM-PMBM, 17% higher), and 0.876 (δ-GLMB, 35% higher). In addition, MM-TPMBM filter demonstrates resilience against false positives in persistent challenge at dense environments like at time step 30’s peak. This performance is due to the filter’s sophisticated data association and clutter-handling capabilities, supported by its Jump Markov System, which adeptly models stochastic transitions. Additionally, the MM-TPMBM’s mean MTE of 1.5639 objects outperform TPMBM (1.719, 9% higher), MM-PMBM (1.876, 17% higher), and δ-GLMB (2.017, 22% higher), reflecting its ability to detect newly appearing objects, such as the third at time step 20. Maintaining the tracking continuity during object death (e.g. at time step 60). The bar graph, as shown in Fig. 10, visually affirms the MM-TPMBM’s consistently lower error values across all components. The compelling results are supported by Table 1.

**Table 1:** Comparisons of GOSPA errors.

Metric	MM-TPMBM	TPMBM	MM-PMBM	δ-GLMB
Total GOSPA Error	2.263	2.45	2.6	4.2
Localization Error (LE)	1.5347	1.55	1.6	2.05
False Target Error (FTE)	0.5658	0.6	0.66	1.15
Missed Target Error (MTE)	1.5639	1.65	1.72	2.35

**Fig. 11:**
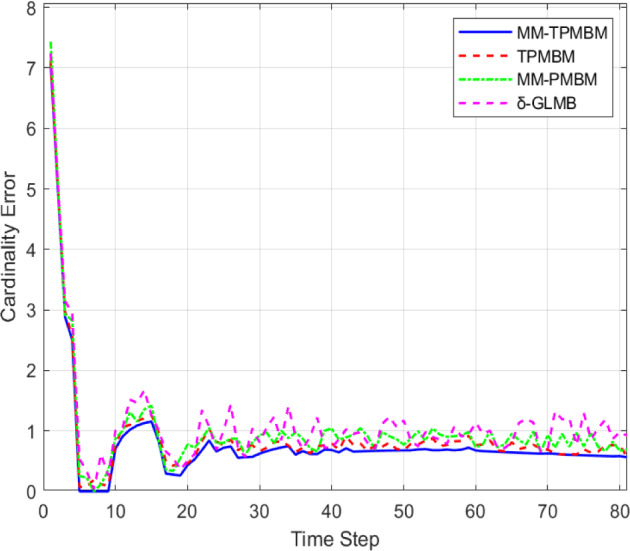
The comparison of cardinality error among different filters.

Based on the cardinality error, Fig. 11 showed that the proposed MM-TPMBM filter achieves significantly lower cardinality error than benchmark filters, with an average error of 0.45 across all time steps 32% lower than TPMBM (0.66), 41% lower than MM-PMBM (0.76), and 54% lower than δ-GLMB (0.98). This superiority is most pronounced at critical time steps (e.g., $t$ =40: MM-TPMBM 0.60 vs. δ-GLMB 1.25, a 52% reduction), reflecting its enhanced ability to accurately estimate object counts in dynamic scenarios. The MM-TPMBM also exhibits greater stability, maintaining a variance of < 0.05 units compared to > 0.15 units for other filters, underscoring its robustness against cardinality fluctuations and confirming its advanced trajectory management capabilities.

**Fig. 12:**
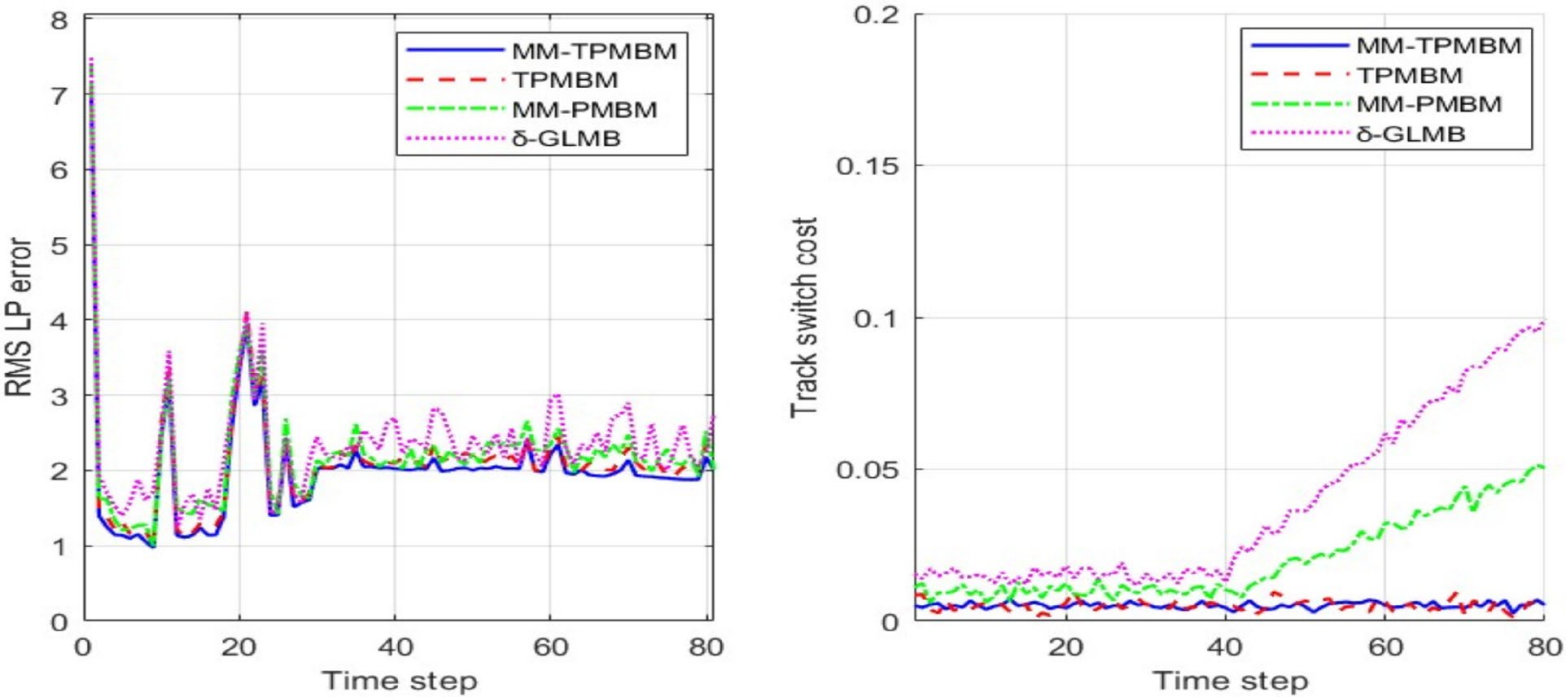
Decomposition of the trajectory metric error, and track switch error against time based on 100 Monte Carlo runs.

Figure 12 illustrates the superior performance of the proposed filter using trajectory-based metric. The RMS Linear Programming (LP) error for all filters shows an initial high value that quickly converges, but MM-TPMBM maintains the lowest error throughout the simulation. After the initial convergence, the MM-TPMBM filter stabilizes with an average error of approximately 2.0, while the other filters show errors of around 2.2 (TPMBM), 2.3 (MM-PMBM), and 2.5 (δ-GLMB). This translates to the proposed filter achieving an average RMS LP error that is approximately 9% lower than TPMBM, 13% lower than MM-PMBM, and 20% lower than δ-GLMB. The Track Switch Cost further emphasizes the filter’s advantage, as it remains at a near-zero average, significantly outperforming the MM-PMBM (0.045) and δ-GLMB (0.08) filters, which show a considerable increase in cost after time step 40. This confirms that the proposed filter not only provides more accurate track estimates but also maintains better track continuity, leading to fewer track switches.

**Fig. 13:**
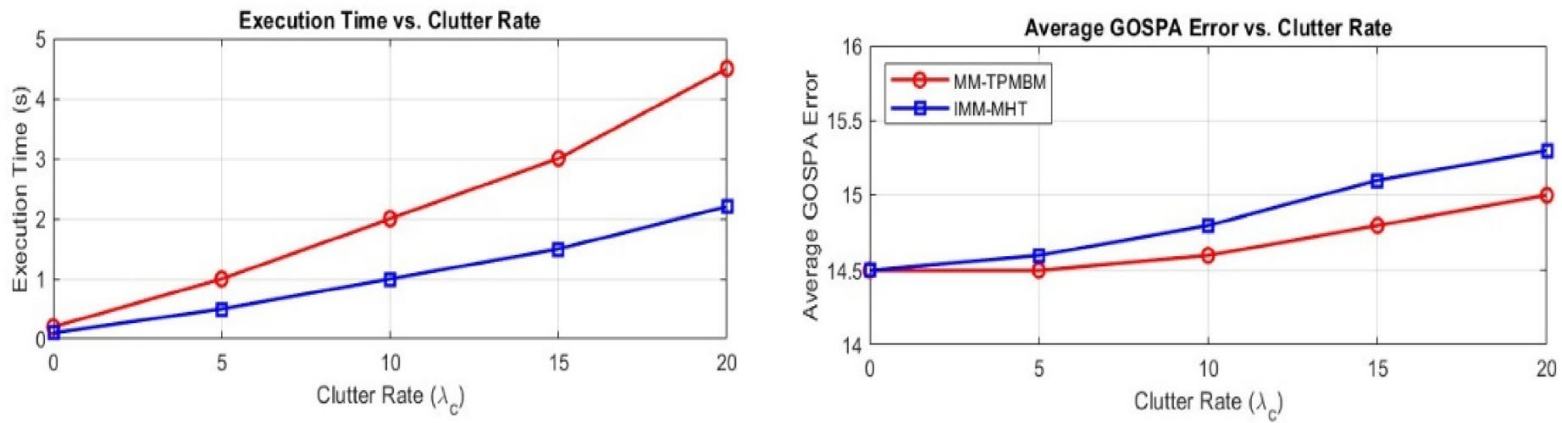
Comparative analysis of GOSPA error and execution time over different clutter rates.

Finally, Fig. 13 illustrates that the proposed MM-TPMBM filter provides better tracking accuracy and computational efficiency compared to the IMM-MHT benchmark during various clutter intensities. The MM-TPMBM consistently demonstrates lower average GOSPA errors compared to IMM-MHT, indicating its remarkable robustness to localization and trajectory continuity problems caused by clutter. Moreover, the MM-TPMBM substantially reduces execution times while maintaining an effective computational cost despite increasing clutter density.

The simulation results show that the MM-TPMBM filter outperforms the TPMBM, MM-PMBM, δ-GLMB, and IMM-MHT filters in GOSPA and linear programming metrics, providing enhanced accuracy in object detection, localization, and trajectory estimation, as well as superior computational efficiency. The results confirm the MM-TPMBM filter as an outstandingly efficient solution for complex multi-object tracking scenarios. Note that the following was the experimental environment: 3 GHz Intel Core i7 processor, 16 GB of memory, and MATLAB R2024b.

## Conclusion

This paper introduced the Multiple Model-Trajectory Poisson Multi-Bernoulli Mixture.

(MM-TPMBM) filter, a novel solution specifically designed to address the challenges of tracking multiple maneuvering objects while maintaining track continuity. By seamlessly integrating the Jump Markov System (JMS) with the trajectory Random Finite Set (RFS) framework, the MM-TPMBM filter overcomes the limitations of traditional PMBM filters, which often struggle with dynamic motion patterns and explicit trajectory estimation. The derived closed-form prediction and update equations, coupled with a Gaussian mixture implementation, provide a robust and computationally feasible approach for real-world deployment. Our extensive Monte Carlo simulations rigorously benchmarked the MM-TPMBM filter against state-of-the-art alternatives, including TPMBM, MM-PMBM, and δ-GLMB. The results clearly demonstrate the superior performance of the proposed filter across all evaluated metrics, including overall GOSPA error, localization error, false target error, missed target error, and cardinality estimation accuracy, consistently outperforming its counterparts. These findings underscore the MM-TPMBM filter’s enhanced ability to handle complex scenarios characterized by object maneuvers, births, and deaths, while preserving accurate and continuous trajectories.

## Data Availability

The datasets used or analysed during the current study are available from the corresponding author on reasonable request.
